# A key to the genera and species of the transversely-dividing Flabellidae (Anthozoa, Scleractinia, Flabellidae), with a guide to the literature, and the description of two new species

**DOI:** 10.3897/zookeys.562.7310

**Published:** 2016-02-10

**Authors:** Stephen D. Cairns

**Affiliations:** 1 Department of Invertebrate Zoology, National Museum of Natural History, Smithsonian Institution, Washington, DC 20560, USA

**Keywords:** Flabellidae, Truncatoflabellum, Placotrochides, Blastotrochus, Placotrochus, Falcatoflabellum, transversely dividing, key, asexual reproduction

## Abstract

The transversely-dividing flabellids consist of five genera (*Truncatoflabellum*, *Placotrochides*, *Blastotrochus*, *Placotrochus*, and *Falcatoflabellum*) and 45 species. A dichotomous key is provided for these five genera as well as the species of the genus *Truncatoflabellum* and *Placotrochides*, the other three genera being monotypic. A tabular key is also provided for the 38 species of *Truncatoflabellum*. Two new combinations are suggested (*Truncatoflabellum gambierense* and *Truncatoflabellum sphenodeum*) and two new species are described (*Truncatoflabellum duncani* and *Truncatoflabellum mozambiquensis*). All but one species are illustrated and accompanied by their known distribution and a guide to the pertinent literature for the species. New records of 19 of the 45 species are listed. The transversely-dividing flabellids range from the Middle Eocene to the Recent at depths of 2–3010 m, and constitute 60% of the 65 known extant species of transversely-dividing Scleractinia.

## Introduction

Confronted with a large collection of *Truncatoflabellum* during a recent (2014) trip to Taiwan, it became apparent that the literature on the species of this genus was scattered and not well organized. Although there were some keys to the species, they were regional in nature, i.e., Philippine region (Cairns 1989b), southwest Indian Ocean (Cairns and Keller 1993), North Pacific (Cairns 1994), and Western Australia (Cairns 1998). No unified key or comparative table existed to update that of Cairns (1989b). *Truncatoflabellum* is the seventh largest Holocene genus among the approximately 240 living scleractinian genera, and thus a key to the species and guide to the pertinent literature was thought appropriate. (The most specious Holocene scleractinian genera are (Hoeksema and Cairns 2015): *Acropora* – about 120 living species, *Caryophyllia* – 75 species, *Balanophyllia* – 59 species, *Montipora* – about 56 species, *Flabellum* – 42 species, *Porites* – about 41 species, and then *Truncatoflabellum*, with 32 living species). The four other truncate flabellid genera are included for completeness: *Blastotrochus*, *Placotrochides*, *Placotrochus*, and *Falcatoflabellum*. Of the 120 living azooxanthellate genera (Roberts et al. 2009), 17 of them (14.2% of the genera) and 65 of the approximately 725 azooxanthellate species (or 9.0% of the species) represent transversely-dividing species: *i.e*., the five flabellid genera previously listed and: *Anthemiphyllia* (in part), *Australocyathus*, *Bourneotrochus*, *Caryophyllia* (in part), *Dunocyathus*, *Endopachys*, *Idiotrochus*, *Kionotrochus*, *Notophyllia*, *Peponocyathus*, *Trochocyathus* (in part), and *Truncatoguynia*.

*Fossil Truncatoflabellum*: Because of the easily diagnosed aspect of the anthocyathus basal scar, fossil *Truncatoflabellum* are easily distinguished, even though most have been attributed to the genus *Flabellum*. Most fossil flabellids cannot be correlated to Recent species, but on the other hand, several have been described as discrete species. The earliest record of a transversely-dividing fossil flabellid was that of Duncan (1864), who reported three truncate species from southern Australia: *Flabellum victoriae* (=*Truncatoflabellum victoriae*) from Muddy Creek (Middle Miocene), Victoria; *Flabellum gambierense* (=*Truncatoflabellum gambierense*) from Mount Gambier, S. Australia (Middle Miocene), and *Flabellum candeanum* (=*Truncatoflabellum duncani*, herein) from the “Murray Tertiaries”, Victoria; these specimens are deposited in the BM. These records were reiterated by Duncan (1870), with the slight addition of several more specimens. Tenison-Woods (1878b) reported additional fossil records of *Flabellum gambierense* and *Flabellum victoriae* from Cape Otway, Victoria (Middle Miocene) and Muddy Creek, Victoria (Middle Miocene), respectively. Specimens from that paper were deposited primarily in the Macleayan Museum, Sydney. The first fossil *Truncatoflabellum* from New Zealand were reported by Tenison-Woods (1880) from the Upper Oligocene Pareora beds: *Truncatoflabellum corbicula*, *Truncatoflabellum sphenodeum*, and *Truncatoflabellum simplex* (types deposited at NZGS, now the GNS). Dennant (1899) added another Miocene species to the Australian fauna, *Flabellum gippslandicum*, from the Gippsland Lake region of Victoria, a species quite similar to *Truncatoflabellum victoriae*. The types from that paper were deposited at the NMV. Gerth (1921) reported three *Truncatoflabellum* from the Lower Miocene to Pliocene of Java, all of which can be related to living species (specimens deposited at the RGM). Umbgrove (1938) reported eight specimens as *Flabellum rubrum* from the Pleistocene of Talaud, Celebes, one of which is *Truncatoflabellum aculeatum*, four of which are unidentifiable to species, and three are *Trochocyathus*. Although not illustrated by Umbgrove (1938), these specimens are also deposited at the RGM (35461). Umbgrove (1950) also reported two *Truncatoflabellum* species from the Lower Pleistocene of the Putjangan Beds of Java, part of one of which has been re-identified as *Truncatoflabellum carinatum* (see Cairns, 1989b). Those specimens were deposited at the Institute of Mines at Delft in 1989. Various species of *Truncatoflabellum* from the Pliocene of Taiwan and Plio-Pleistocene of the Ryukyu Islands were reported by Yabe and Eguchi (1942a, b) under the rubric of *Flabellum rubrum*. Most of these specimens, deposited at the TIUS, were examined by the author and re-identified in Cairns (1989b). Yabe and Eguchi (1941) also reported one fossil *Truncatoflabellum* (=*Truncatoflabellum spheniscus*) from the “Neogene” of Java. Squires (1958: pl. 12, figs 6-7) illustrated two *Truncatoflabellum* from the Altonian (Lower Miocene) of New Zealand as *Flabellum rubrum rubrum*, but these are certainly not *Flabellum rubrum* and have not been subsequently re-identified. Most specimens from that paper are deposited at the AUC. Hayward reported *Flabellum sphenodeum* (=*Truncatoflabellum sphenodeum*) from the Lower Miocene of North Auckland, New Zealand; these specimens are also deposited at the AUC. Wells (1984) reported two species from the Late Pleistocene deposits of Kere River, Santo, Vanuatu: *Flabellum vanuatu* (=*Truncatoflabellum vanuatu*) and *Flabellum paripavoninum* (=*Truncatoflabellum mortenseni*); these specimens are deposited at the NMNH. Finally, Hu (1987) reported two truncate flabellids from the Maanshan Mudstone (Plio-Pleistocene) of Hengchun Peninsula, southern Taiwan: *Flabellum transversale* (=*Truncatoflabellum carinatum*) and *Flabellum elongatum* (=*Placotrochides scaphula*); these specimens are deposited at the National Museum of Natural Science, Taichung, Taiwan, and seen by the author in 2014. Hu (1988) also reported *Flabellum rubrum stokesii* from the Tunghsiao and Lungkang Pleistocene formations of the Miaoli District, northern Taiwan, some of which are probably *Truncatoflabellum carinatum*.

It should be noted that in a comprehensive phylogenetic analysis of the Scleractinia using the CO1 gene (Kitahara et al. 2010), in which 65 additional deep-water species were included to the data base, *Truncatoflabellum* was found to be polyphyletic and always ancestral to species within the genus *Flabellum*. Both genera have their earliest records in the Eocene.

## Methods

This is not a taxonomic revision or a phylogenetic or morphometric analysis. It is a key to facilitate identification of a species-rich group, accompanied with a guide to the literature. The synonymies are not exhaustive, but include the original description and those papers that were found useful in identification of the species, especially those that contain useful illustrations, descriptions and/or extended synonymy. Furthermore, the key incorporates exclusively fossil species that occur in the respective genera. Since the key is intended to serve a practical purpose and include fossil species, molecular sequencing was not employed.

In an effort to discuss and illustrate morphologically similar species in adjacent text, and to facilitate their identification through keys, the text and illustrations are arranged in the order in which they occur in the key.

**Figure 1.**
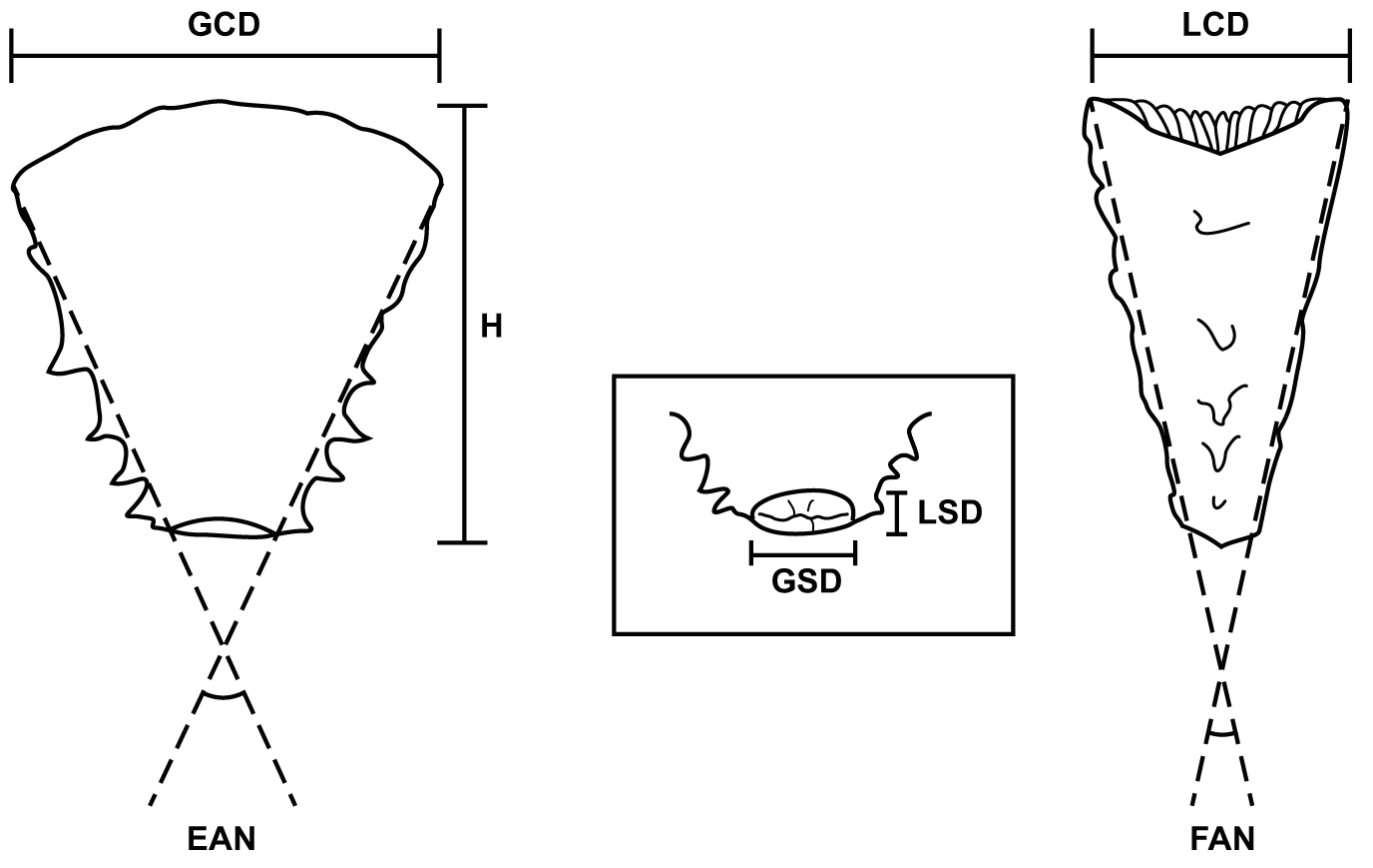
Diagram showing abbreviations of geometric terms used to describe truncate flabellids. Left: lateral view of an anthocyathus; center: basal scar of an anthocyathus; right: edge view of an anthocyathus. Abbreviations defined in Materials section.

The key is based primarily on the morphology of the (free-living) anthocyathus stage of each species, the founding (attached) anthocaulus stage rarely being collected and usually of generic morphology. The shape of the anthocyathus contains the primary distinguishing set of characters for these genera, the shape most accurately defined by the thecal edge and face angles (Fig. 1). These two measurements geometrically define the GCD:LCD, and thus that index is not an independent one, but is presented in Table 2 because of its ease in visualization. The H:GCD is a general measure of the height of the corallum, but is dependent on the size (maturity) of the corallum, thus adult specimens are best measured for this characteristic. The maximum greater scar diameter (GSD), on the other hand, is fairly constant, being the same size for juvenile or large specimens; however, the ratio of GSD:GCD is dependent on the size of the corallum. In addition to shape criteria, the number of pairs of thecal edge spines seems to be relatively constant, some species having none, others one basal pair, others four or more pairs, and still others two or three pairs. The purpose of the thecal edge spines is unknown, however Tokuda et al. (2010) suggest that they function to stabilize “the life position” of the anthocyathus after transverse division. Several species have crests instead of spines. Other characters useful in differentiating species are: number and symmetry of the septa, nature of the upper outer edges of the septa as they meet the theca (e.g., notched, attenuate, abrupt), and corallum color. Geography, fossil occurrence, and even depth distribution may also be used as circumstantial characters.

Of the 45 species of truncate flabellids, 41 are represented in the NMNH collections, including types of 27 of those species. Of those four species not represented in the NMNH collections, photographs were obtained of three (*Truncatoflabellum inconstans*, *Truncatoflabellum gippslandicum*, and *Truncatoflabellum sphenodeum*); only *Truncatoflabellum trapezoideum* (known only from one specimen deposited in Moscow) was not re-examined and not illustrated herein. Whenever possible, five views of a typical anthocyathus of each species is presented in a vertical arrangement, top to bottom: lateral face, edge, basal scar, calice, and oblique calice.

### Abbreviations used in the text include

AUC
 Auckland University College (Dept. of Geology), New Zealand 


BM
 British Museum, London (The Natural History Museum) 


EAN
 Edge Angle: angle formed by two lateral edges of an anthocyathus  (Fig. 1)

FAN
 Face Angle: angle formed by two faces of an anthocyathus  (Fig. 1)

GCD
 Greater calicular diameter of anthocyathus  (Fig. 1)

GCD:LCD
 Ratio of greater calicular diameter to lesser calicular diameter of an anthcyathus 


GNS
 Institute of Geological and Nuclear Sciences, Lower Hutt, New Zealand 


GSD
 Greater scar diameter of anthocyathus  (Fig. 1)

GSD:GCD
 Ratio of basal greater scar diameter to greater calicular diameter of anthocyathus 


H
 Height of corallum  (Fig. 1)

H:GCD
 Ratio of corallum height to greater calicular diameter  (Fig. 1)

IOM
 Institute of Okeanology, Moscow 


IWP
 Indo-West Pacific 


LCD
 Lesser calicular diameter of anthocyathus  (Fig. 1)

LSD
 Lesser scar diameter of anthocyathus  (Fig. 1)

NMNH
 National Museum of Natural History, Smithsonian Institution, Washington, DC 


NMV
 National Museum of Victoria, Victoria, Australia 


NZGS
 New Zealand Geological Survey (now the GNS), Lower Hutt, New Zealand 


RGM
National Museum of Geology and Mineralogy (at present Naturalis Biodiversity Center, Leiden) 


SAM
South African Museum, Cape Town 


SIPHILEXP
Smithsonian Institution Philippines Expedition 


SWIO
 Southwest Indian Ocean 


Sx, Cx, Px
 Cycle of septa, costae, or pali, respectively, designated by numerical subscript 


Sx>Sy
 In the context of a septal formula, septa of cycle x are wider than those of cycle y


Sx°>Sy° In the context of a septal formula, septa size class x are wider than those of size class y


TIUS
Institute of Geology and Paleontology, Tohoku (Imperial) University, Sendai, Japan 


USNM
 United States National Museum (now the NMNH) 


### Key to the Transversely-Dividing Flabellid Genera

**Table:** 

1	Columella rudimentary (trabecular) or absent	**2**
1’	Columella lamellar or fascicular	**4**
2	Anthocyathus buds only from a basal anthocaulus	**3**
2’	Anthocyathi bud from basal anthocaulus (transverse division) and from lateral edges of anthocaulus (forming anthoblasts)	***Blastotrochus*** (1 species)
3	Anthocyathus usually fan-shaped with divergent thecal edges, but if compressed-cylindrical in shape, the latter bear edge spines; base of anthocaulus not stereome-reinforced	***Truncatoflabellum*** (38 species)
3’	Anthocyathus compressed-cylindrical in shape (edge angle 0-5°), lacking lateral spines; base of anthocaulus stereome-reinforced	***Placotrochides*** (4 species)
4	Columella lamellar	***Placotrochus*** (1 species)
4’	Columella fascicular	***Falcatoflabellum*** (1 species)

## Guide to the literature, distribution, and remarks

### Family Flabellidae Bourne, 1905

#### Genus Truncatoflabellum

Taxon classificationAnimaliaScleractiniaFlabellidae

Cairns, 1989b

Flabellum : Milne Edwards and Haime 1848: 257, 259 (in part: *flabelline tronquees*).—Vaughan and Wells 1943: 226-227 (in part).—Wells 1956: F432 (in part).—Zibrowius 1974: 19 (in part: group 2, but not *Blastotrochus nutrix*).
Truncatoflabellum Cairns, 1989b: 60–61; 1994: 75; 1995: 113.—Cairns and Kitahara 2012: 14 (key to genus).

##### Diagnosis.

Asexual reproduction by apical transverse division of corallum, resulting in distal anthocyathus and basal anthocaulus. Corallum usually laterally compressed and fan shaped, having one or more pairs of thecal edge spines or crests; some species compressed-cylindrical in shape but these always laterally spinose, whereas some fan-shaped coralla lack spines and crests. Columella absent or represented by a fusion of the lower, axial edges of larger septa. Anthocaulus not stereome-reinforced.

##### Discussion.

The taxonomic history of this genus extends long before it was officially described, and is recounted and discussed by Cairns (1989b). To briefly reiterate, even as early as 1848 Milne Edwards and Haime (1848) placed these species in a section (=subgenus) they called the “*flabelline tronquees*”. Squires (1963: 10, 25) strongly felt that this group of species should be separated as a genus different from *Flabellum* but ultimately did not take an action, waiting for more biological justification. In Zibrowius’ (1974) revision of the family Flabellidae, he placed the transversely-dividing *Flabellum* as one of three “groups” in the larger conventional genus *Flabellum*. Finally, in a paper about the various modes of asexual reproduction, Cairns (1989a) suggested that transverse division represented a key innovation that led to an adaptive advantage for living on soft substrates, justifying the naming of a new genus. But, it was not until later in that year that Cairns (1989b) proposed the name *Truncatoflabellum*. As of this paper, there are 38 known species in the genus, six of these known only as fossils (Table 1).

**Table 1.** Transversely dividing flabellids, arranged by predominant geographic region (+ = fossil).

**Philippines and Indonesia**
***Truncatoflabellum*** Cairns, 1989 (38 spp, including 6 exclusively fossil)
***compressum*** (Lamarck, 1816)
=*stokesii* (Milne Edwards & Haime, 1848)
=*Flabellum oweni* Milne Edwards & Haime, 1848
***spheniscus*** (Dana, 1846)
=*Flabellum debile* Milne Edwards & Haime, 1848
=*Flabellum affine* Milne Edwards & Haime, 1848
=*Flabellum bairdi* Milne Edwards & Haime, 1848
=*Flabellum profundum* Milne Edwards & Haime, 1848
=*Flabellum sumatrense* Milne Edwards & Haime, 1848
=*Flabellum crenulatum* Milne Edwards & Haime, 1848
=*Flabellum elongatum* Milne Edwards & Haime, 1848
=+*variabile* *sensu* Gerth, 1921 (new synonymy)
***aculeatum*** (Milne Edwards & Haime, 1848)
=?*Flabellum spinosum* Milne Edwards & Haime, 1848
=?*Flabellum variabile* Semper, 1872
***crassum*** (Milne Edwards & Haime, 1848)
***candeanum*** (Milne Edwards & Haime, 1848)
=*Flabellum elegans* Milne Edwards & Haime, 1848
***cumingi*** (Milne Edwards & Haime, 1848)
=*Flabellum irregulare* Tenison-Woods, 1878: 313 (junior homonym of Semper’s 1872, but no need of new name since it is a junior synonym)
***irregulare*** (Semper, 1872)
***paripavoninum*** (Alcock, 1894)
***dens*** (Alcock, 1902)
***incrustatum*** Cairns, 1989
=+*irregulare* *sensu* Gerth, 1921:402 (new synonymy)
***formosum*** Cairns, 1989
=*Truncatoflabellum* sp. n. *sensu* Cairns, 1989:73
***pusillum*** Cairns, 1989
***carinatum*** Cairns, 1989
?+***variablealta*** Gerth, 1921, if so, name is ***altum***
***angustum*** Cairns & Zibrowius, 1997
**Central and eastern Pacific**
***trapezoideum*** (Keller, 1981)
***truncum*** (Cairns, 1982)
**Vanuatu, Wallis andFutuna, New Caledonia**
***martensii*** (Studer, 1878)
=+**paripavoninum** *sensu* Wells, 1984
***mortenseni*** Cairns & Zibrowius, 1997
***vanuatu*** (Wells, 1984)
***vigintifarium*** Cairns, 1999
**New Zealand and Kermadecs**
***arcuatum*** Cairns, 1995
***phoenix*** Cairns, 1995
=*Truncatoflabellum* sp. B *sensu* Cairns, 1994
**Western Australia**
***angiostomum*** (Folkeson, 1919)
***australiensis*** Cairns, 1998
***veroni*** Cairns, 1998
***macroeschara*** Cairns, 1998
**Western Indian Ocean/S. Africa**
***stabile*** (Marenzeller, 1904)
=Truncatoflabellum sp. A *sensu* Cairns, 1994: 79
=?*Truncatoflabellum* sp. Zibrowius & Gili, 1990
***inconstans*** (Marenzeller, 1904)
***gardineri*** Cairns in Cairns & Keller, 1993
***zuluense*** Cairns in Cairns & Keller, 1993
***multispinosum*** Cairns in Cairns & Keller, 1993
***mozambiquensis*** sp. n.
**South Australian exclusively fossil species**
+***victoriae*** (Duncan, 1864)
=?*Flabellum simplex* Tenison-Woods, 1878
+***gambierense*** (Duncan, 1864) (new combination)
+***corbicula*** (Tenison-Woods, 1880)
+***sphenodeum*** (Tension Woods, 1880) (new comb.)
+?*Flabellum attenuatum* Tenison-Woods, 1880
+***gippslandicum*** (Dennant, 1899)
+***duncani*** sp. n.
=***candeanum*** *sensu* Duncan 1870
***Blastotrochus*** Milne Edwards & Haime, 1848
***nutrix*** Milne Edwards & Haime, 1848
+***proliferus*** d’Archiardi, 1866 (= ?*Cladocora*)
***Placotrochides*** Alcock, 1902
***scaphula*** Alcock, 1902
=+*Flabellum elongatum* Hu, 1987 (junior homonym of ME and H, 1848)
***frustum*** Cairns, 1979
***cylindrica*** Cairns, 2004
***minuta*** Cairns, 2004
=*minima* (*lapsus calumni*) *sensu* Cairns, 2006
***Placotrochus*** Milne Edwards & Haime, 1848
***laevis*** Milne Edwards & Haime, 1848
=*Placotrochus candeanus* Milne Edwards & Haime, 1848
=*Placotrochus pedicellatus* Tenison-Woods, 1879
***Falcatoflabellum*** Cairns, 1995
***rauolensis*** Cairns, 1995

##### Distribution.

Middle Eocene (Bortonian) of New Zealand to Recent: cosmopolitan, except for the Antarctic, northeast Pacific and western Atlantic (generally low species diversity in Atlantic), 2–3010 m.

##### Type species.

*Euphyllia spheniscus* Dana, 1846, by original designation.

### Key to the species of *Truncatoflabellum* (characteristics pertain to the anthocyathus stage unless otherwise stated; + exclusively fossil species)

**Table:** 

1	One or more pairs of thecal edge spines present	**2**
1’	Thecal edge spines not present	**28**
2	Corallum compressed-cylindrical (edge angle 0–15°)	**3**
2’	Corallum compressed-conical or fan-shaped (edge angle >15°)	**5**
3	Corallum small (GCD < 4.5 mm); rejuvenescence common, resulting in a high H:GCD (e.g., up to 4.3); corallum brown; 32 or less septa	***Truncatoflabellum phoenix*** (Fig. 2A)
3’	Corallum larger (GCD >10 mm); rejuvenescence not common (H:GCD = 1–2); corallum white; 48 or more septa	**4**
4	Corallum with more than 48 septa (*e.g.*, 76)	+***Truncatoflabellum gippslandicum*** (Fig. 2B)
4’	Corallum with 48 septa	+***Truncatoflabellum victoriae*** (Fig. 2C)
5	GCD < 12 mm	**6**
5’	GCD > 12 mm	**9**
6	Tendency for anthocyathus to remain attached to anthocaulus	**7**
6’	Anthocyathus and anthocaulus always detached	**8**
7	Thecal face angle low (14–18°), resulting in a high GCD:LCD (1.7–2.3); bimodal edge angle; IWP in distribution	***Truncatoflabellum dens*** (Fig. 2D)
7’	Face angle higher (18–22°), resulting in a lower GCD:LCD (1.4–1.8); angle of thecal edges not bimodal; SWIO	***Truncatoflabellum zuluense*** (Fig. 3A)
8	Thecal edge angle low (14–18°), resulting in a small GCD:LCD (e.g., 1.4–1.7)	***Truncatoflabellum pusillum*** (Fig. 3B)
8’	Thecal edge angle higher (28–52°), resulting in a higher GCD:LCD (e.g., 1.85–2.3)	***Truncatoflabellum angustum*** (Fig. 3C)
9	One (basal) pair of thecal edge spines present	**10**
9’	Two or three pairs of thecal edge spines present	**19**
9’’	Four of more pairs of thecal edge spines present	**26**
10	Thecal edge angle >80°; upper calicular edge strongly arched; S7 often present	**11**
10’	Thecal edge angle 15–80°; calicular edge not strongly arched; S7 never present	**13**
11	Basal scar quite small (less than 4.3 mm in length), GSD:GCD < 0.1	***Truncatoflabellum angiostomum*** (Fig. 3D)
11’	Basal scar large (up to 30 mm in length), GSD:GCD = 0.35–0.55	12
12	Thecal edge angle small (55–85°); GCD:LCD = 2.5–3.1	***Truncatoflabellum macroeschara*** (Fig. 4A)
12’	Thecal edge angle larger (95–127°); GCD:LCD = 3.0–4.8	***Truncatoflabellum veroni*** (Fig. 4B)
13	H:GCD > 1; thecal edge angle 15–30°	14
13’	H:GCD <1; thecal edge angle 30–80°	**17**
14	Anthocaulus and anthocyathus remain attached to each other; anthocaulus elongate, narrow, and often bent; Miocene of S. Australia and Victoria	+***Truncatoflabellum gambierense*** (Fig. 4C)
14’	Anthocaulus and anthocyathus detach from each other; anthocaulus not elongate; Recent of IWP	**15**
15	Septa hexamerally arranged in three or four size classes (S1–2>S2>S4>S5); upper outer septal margin not notched	**16**
15’	Septa arranged in three size classes, but not hexamerally (e.g., 16–18: 16–18: 32–36); upper outer septal margin slightly notched	***Truncatoflabellum irregulare*** (Fig. 4D)
16	Scar diameter up to 10 mm; Lower Miocene to Recent	***Truncatoflabellum incrustatum*** (Fig. 5A)
16’	Scar diameter less than 4 mm; Middle Eocene to Middle Miocene	+***Truncatoflabellum sphenodeum*** (Fig. 5B)
17	GSD:GCD <0.3	***Truncatoflabellum crassum*** (Fig. 5C)
17’	GSD:GCD >0.3	**18**
18	Corallum white; GSD up to 15 mm	***Truncatoflabellum aculeatum*** (Fig. 5D)
18’	Corallum striped reddish-brown; GSD less than 7 mm	***Truncatoflabellum mortenseni*** (Fig. 6A)
19	Calicular margin scalloped	**20**
19’	Calicular margin straight (not scalloped)	**21**
20	Basal scar large (up to 8.6 in GSD); thecal face angle low (18–28°), resulting in a large GCD:LCD (1.9–2.4); Western Australia	***Truncatoflabellum australiensis*** (Fig. 6B)
20’	Basal scar smaller (less than 5.7 mm in GSD); thecal face angle higher (30–41°), resulting in a lower GCD:LCD (1.6–2.0); IWP	***Truncatoflabellum candeanum*** (Fig. 6C)
21	Septal symmetry hexameral, up to sixth cycle	**22**
21’	Septal symmetry not hexameral, but in three size classes	**24**
22	Basal scar large (up to 13.7 mm in length); GSD:GCD > 0.35; theca white	***Truncatoflabellum compressum*** (Fig. 6D)
22’	Basal scar smaller (less than 10 mm in length); GSD:GCD < 0.3; theca blackish	**23**
23	GSD:GCD = 0.28–0.30; three pairs of thecal edge spines; thecal edges acute; H:GCD = 0.83–1.0	***Truncatoflabellum martensii*** (Fig. 7A)
23’	GSD:GCD = 0.19–0.26; one (often two) short thecal edge spines; thecal edges rounded; H:GCD = 1.0–1.4	***Truncatoflabellum mozambiquensis*** (Fig. 7B)
24	Septal symmetry in multiples of 20; theca striped reddish-brown; GSD:GCD <0.15	***Truncatoflabellum vigintifarium*** (Fig. 7C)
24’	Septal symmetry in multiples of 16 or 18; theca white; GSD:GCD >0.3	25
25	GCD:LCD = 2.3–3.6 thecal edge angle 82–90°	***Truncatoflabellum spheniscus*** (Fig. 7D)
25’	GCD:LCD = 1.8–2.0; thecal edge angle 31–44°	***Truncatoflabellum cumingi*** (Fig. 8A)
26	Thecal edge angle 41–56°; H:GCD < 1.0; theca brown; axial edges of septa sinuous; SWIO	**27**
26’	Thecal edge angle 20–27°; H:GCD = 1.7–2.0; theca white; central Pacific	***Truncatoflabellum vanuatu*** (Fig. 8B)
27	Upper outer edges of S1–3 attenuate gracefully to meet theca; Miocene of S. Australia	+***Truncatoflabellum duncani*** (Fig. 8C)
27’	Upper outer septal edges not attenuate; Recent of IWP	***Truncatoflabellum multispinosum*** (Fig. 8D)
28	Thecal edges rounded or acute, but never crested	**29**
28’	Thecal edges discontinuously crested	**34**
29	Thecal edge angle = 65–138°; thecal face angle = 32–82°; axial septal edges straight	***Truncatoflabellum paripavoninum*** (Fig. 9A)
29’	Thecal edge angle < 70°; thecal face angle < 38°; axial septal edges sinuous	**30**
30	GSD:GCD < 0.2	**31**
30’	GSD:GCD >0.25	**32**
31	Thecal edge angle 60–90°; H:GCD = 0.7–1.1; deep water (786–3010 m)	***Truncatoflabellum stabile*** (Fig. 9B)
31’	Thecal edge angle 40–50°; H:GCD =1.0–1.5; shallow water (100 m)	***Truncatoflabellum inconstans*** (Fig. 9C)
32	Costae (C1–3) ribbed; thecal edge angle 45–80°	**33**
32’	Costae not ribbed; thecal edge angle less than 20°; fossil from New Zealand	+***Truncatoflabellum corbicula*** (Fig. 9D)
33	H:GCD = 0.9–1.2; C1–3 ribbed; southeastern Pacific	***Truncatoflabellum truncum*** (Fig. 10A)
33’	H:GCD = 0.7; C1–2 ribbed; mid-Pacific	***Truncatoflabellum trapezoideum***
34	Septal symmetry in multiples of 20 (*e.g*., 20: 20: 20: 80)	***Truncatoflabellum formosum*** (Fig. 10B)
34’	Septal symmetry hexameral in four to five cycles	**35**
35	Five cycles of septa and part of sixth; H:GCD <1.2	***Truncatoflabellum carinatum*** (Fig. 10C)
35’	Four cycles of septa and part of fifth; H:GCD >1.3	**36**
36	H:GCD = 1.3–1.9; GCD:LCD = 1.3–1.5	***Truncatoflabellum gardineri*** (Fig. 10D)
36’	H:GCD = 2.9–3.5; GCD:LCD = 1.8–2.6	***Truncatoflabellum arcuatum*** (Fig. 11A)

**Figure 2.**
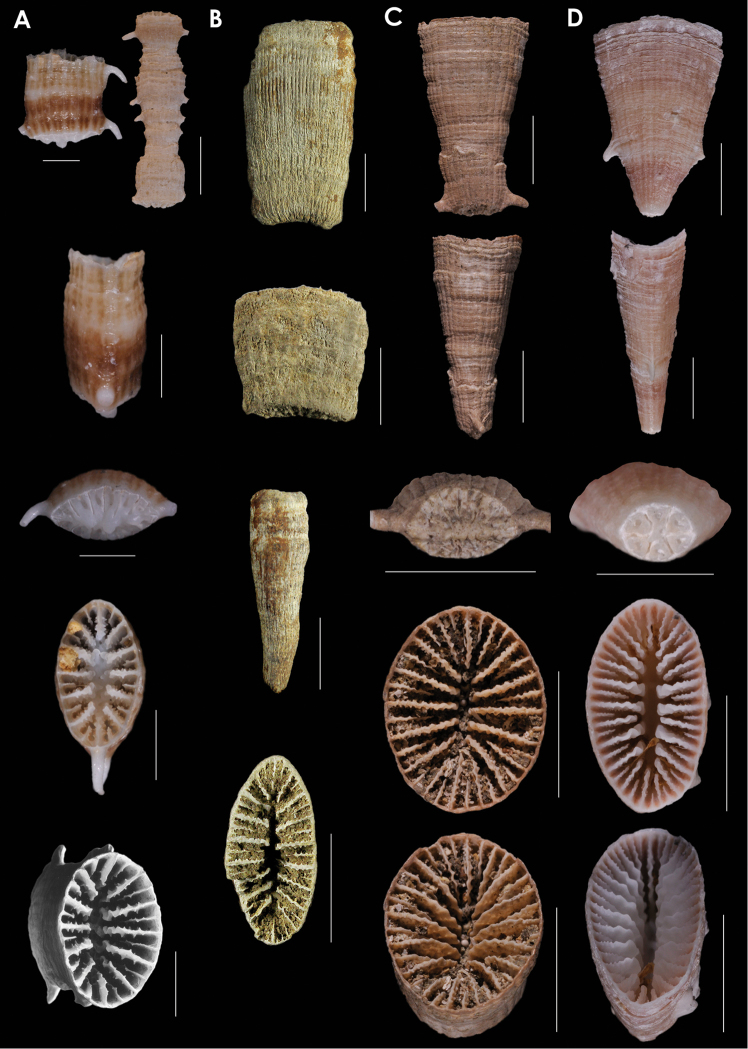
**A** *Truncatoflabellum phoenix*, paratypes, USNM 82010, Kermadec Ridge **B** *Truncatoflabellum gippslandicum*: upper lateral and edge views, NMV P133990; lower lateral and calicular views, syntype, NMV P27064, Miocene of Gippsland lake region of Victoria **C** *Truncatoflabellum victoriae*, USNM 67962, Muddy Creek, Victoria (Balcombian = Middle Miocene) **D** *Truncatoflabellum dens*, USNM 98889, MUSORSTOM 7-569, Vanuatu. Scale bars: 2 mm (**A**); 10 mm (**B**); 5 mm (**C–D**).

**Figure 3.**
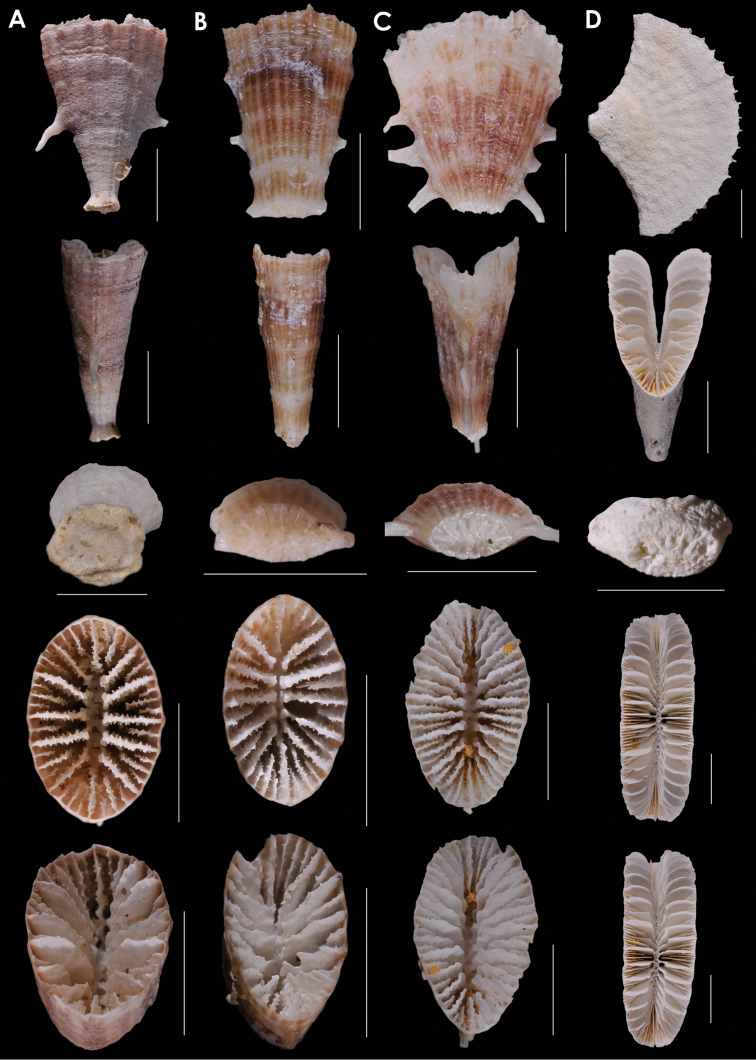
**A** *Truncatoflabellum zuluense*, paratype, USNM 91751, MD ZK-20, South Africa **B** *Truncatoflabellum pusillum*, holotype, USNM 81978, *Albatross* 5178, Philippines **C** *Truncatoflabellum angustum*, USNM 98894, MUSORSTOM 8-1016, Vanuatu **D** *Truncatoflabellum angiostomum*, USNM 96643, Cape Jaubert, Western Australia. Scale bars: all 10 mm, except for basal scar views, which are 5 mm.

**Figure 4.**
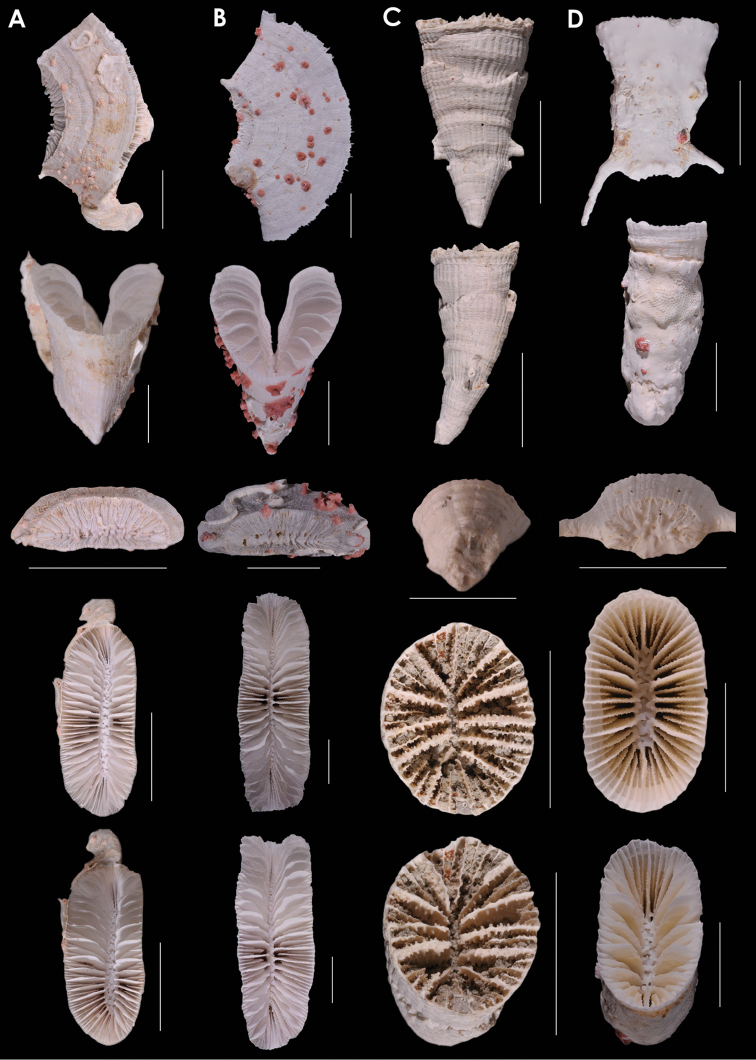
**A** *Truncatoflabellum macroeschara*, paratype, USNM 96661, Onslow Island, Western Australia **B** *Truncatoflabellum veroni*, paratype, USNM 96655, *Soela* 54A, Western Australia **C** *Truncatoflabellum gambierense*, USNM 1295473, USGS 10809, Balcombe’s Bay, Victoria (Balcombian = Middle Miocene) **D** *Truncatoflabellum irregulare*, USNM 87713, Japan. Scale bars: all 10 mm.

**Figure 5.**
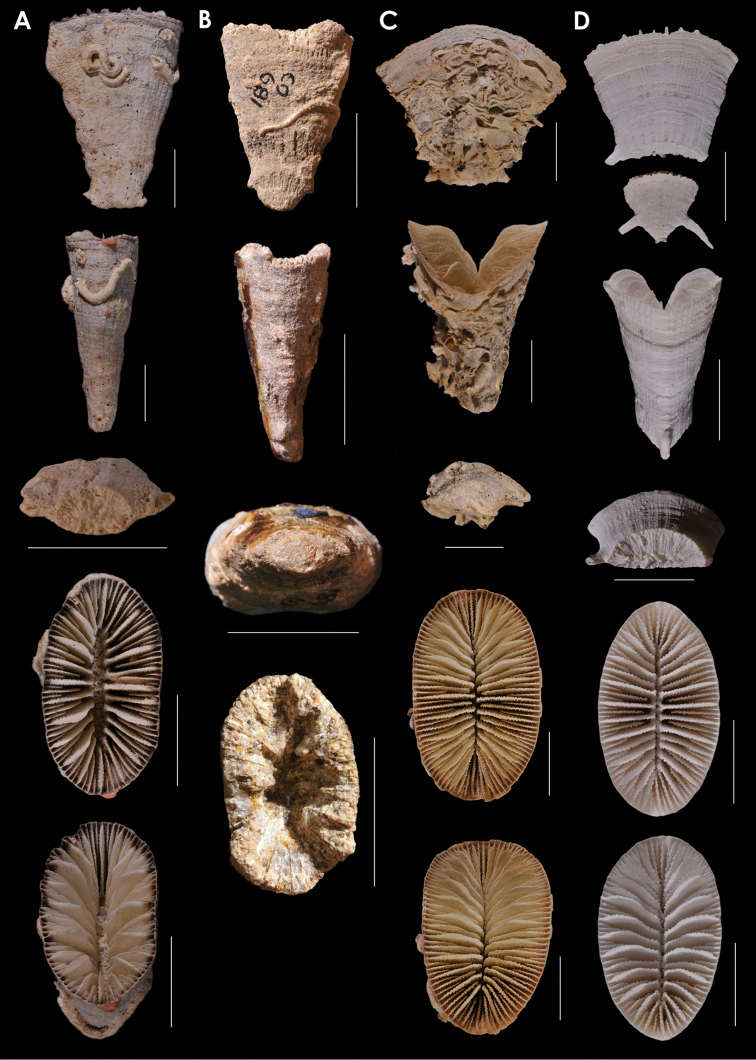
**A** *Truncatoflabellum incrustatum*, holotype, USNM 40774, *Albatross* 5251, Philippines **B** *Truncatoflabellum sphenodeum*, lectotype, NZGS CO 681, Trilissick Basin, New Zealand (Duntroonian = Lower Oligocene) **C** *Truncatoflabellum crassum*, USNM 1130686, *Albatross* 5270, Philippines **D** *Truncatoflabellum aculeatum*, USNM 40781, *Albatross* 5156, Philippines. Scale bars: all 10 mm.

**Figure 6.**
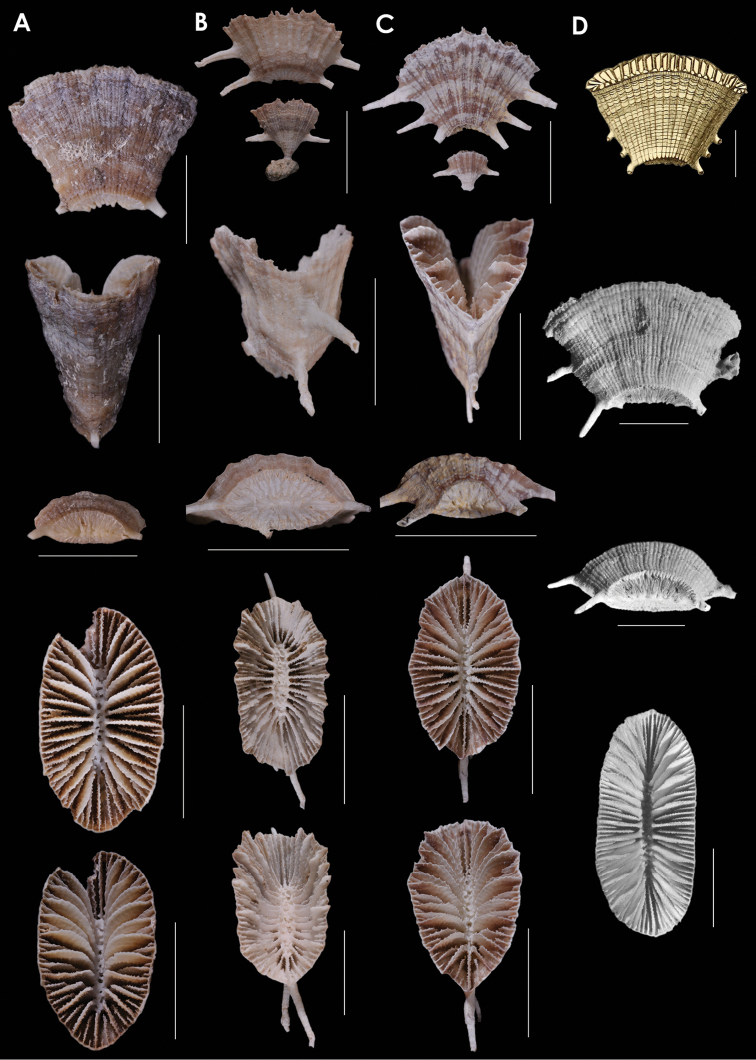
**A** *Truncatoflabellum mortenseni*, USNM 97522, paratype, Philippines **B** *Truncatoflabellum australiensis*, paratype (including anthocaulus), USNM 96652, Western Australia **C** *Truncatoflabellum candeanum*, neotype, including anthocaulus, USNM 81963, *Albatross* 5369, Philippines **D** *Truncatoflabellum compressum*, upper figure, illustration of type from Lesson (1827); other views from *Challenger* 190, BM 1880.11.25.78. Scale bars: all 10 mm.

**Figure 7.**
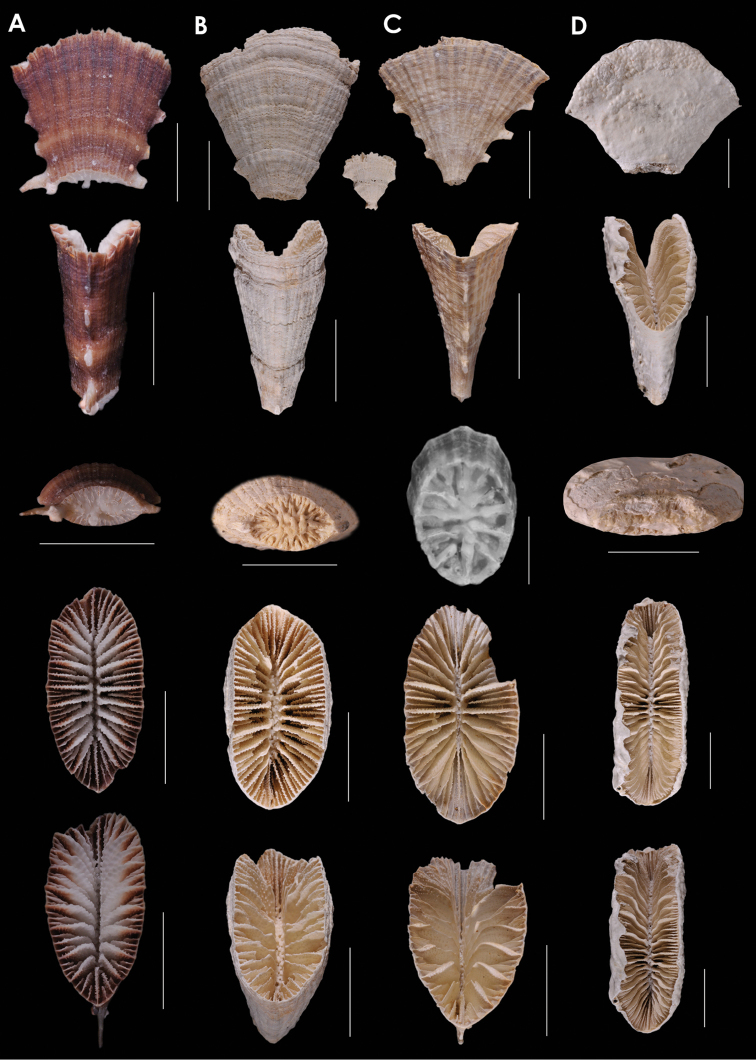
**A** *Truncatoflabellum martensii*, USNM 98908, MUSORSTOM 8 1085, Vanuatu **B** *Truncatoflabellum mozambiquensis*, holotype, USNM 91764, and anthocaulus, *Anton Bruun* 372L, Mozambique **C** *Truncatoflabellum vigintifarium*, paratype, USNM 98900, MUSORSTOM 1018, Vanuatu **D** *Truncatoflabellum spheniscus*, syntype, USNM 92, Singapore. Scale bars: all 10 mm, except for basal scar of C, which is 5 mm.

**Figure 8.**
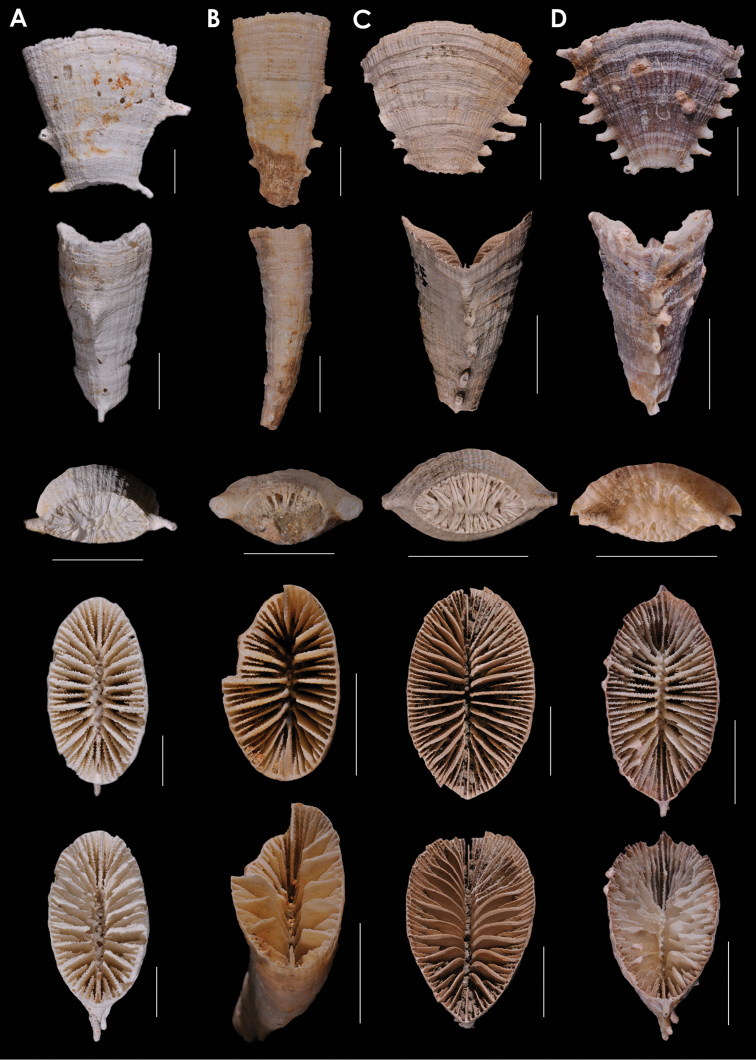
**A** *Truncatoflabellum cumingi*, neotype, USNM 81976, *Te Vega* 1-54, Indonesia **B** *Truncatoflabellum vanuatu*, holotype, USNM 71860, Pleistocene of Vanuatu **C** *Truncatoflabellum duncani*, paratype, USNM M353592, Balcombe’s Bay, Victoria (Balcombian = Middle Miocene) **D** *Truncatoflabellum multispinosum*, paratype, USNM 91741, South Africa. Scale bars: all 10 mm, except for basal scar views of **B** and **C**.

**Figure 9.**
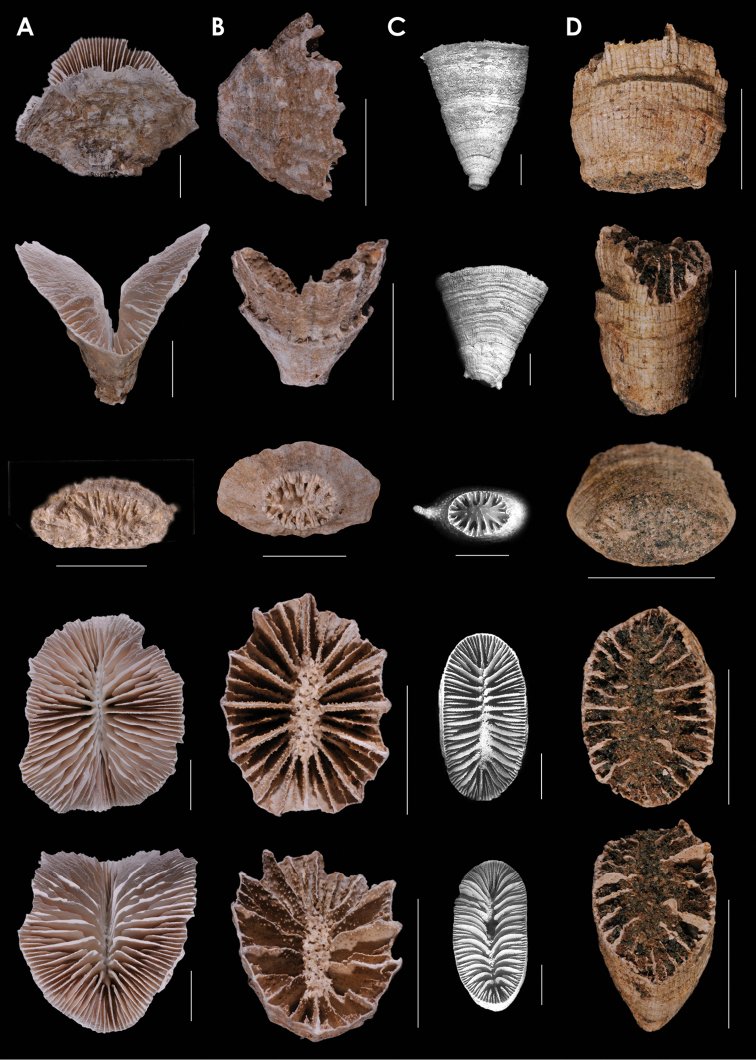
**A** *Truncatoflabellum paripavoninum*, USNM 96650, *Soela* 1/84/77, Western Australia **B** *Truncatoflabellum stabile*, USNM 98886, off Madeira **C** *Truncatoflabellum corbicula*, USNM 67939, NZGS GS1341, Waitaki Valley, New Zealand (Duntroonian = Lower Oligocene) **D** *Truncatoflabellum inconstans*, syntypes, *Valdivia* 100, Zoologisches Museum Berlin. Scale bars: all 10 mm, except for basal scar views of **B** and **C**.

**Figure 10.**
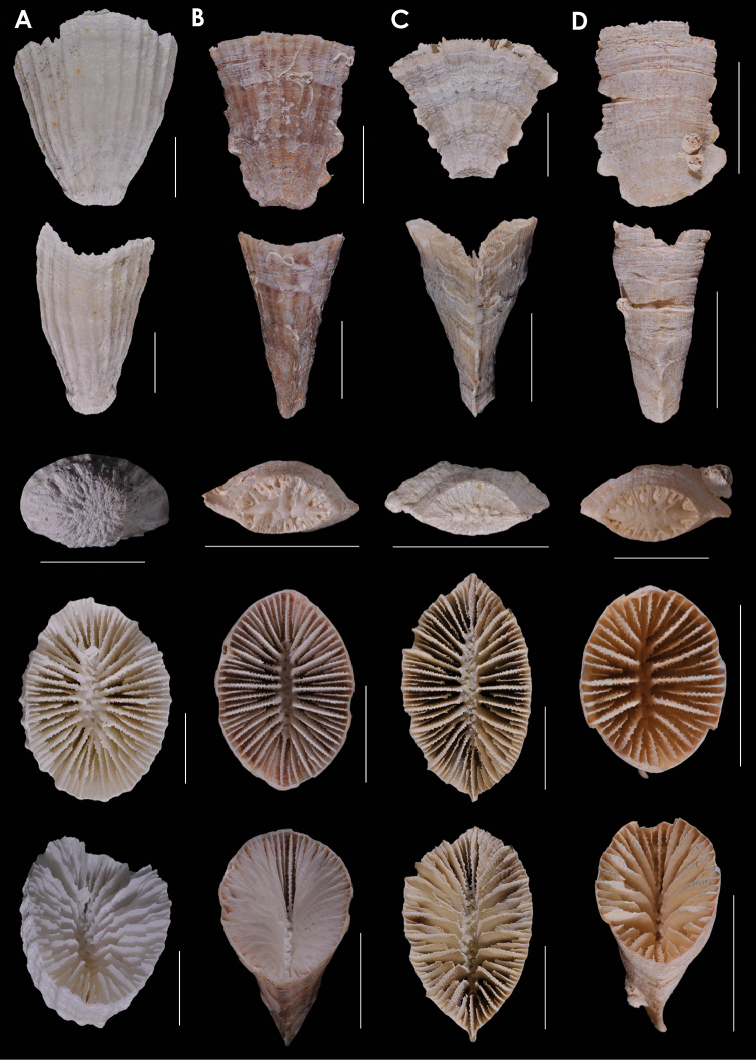
**A** *Truncatoflabellum truncum*, holotype, *Eltanin* 283, Strait of Magellan **B** *Truncatoflabellum formosum*, USNM 91757, *Vityaz* 2635, off Mozambique **C** *Truncatoflabellum carinatum*, USNM 92806, Taiwan **D** *Truncatoflabellum gardineri*, USNM 91736, holotype, *Anton Bruun* 7-3905, South Africa. Scale bars: all 10 mm.

**Figure 11.**
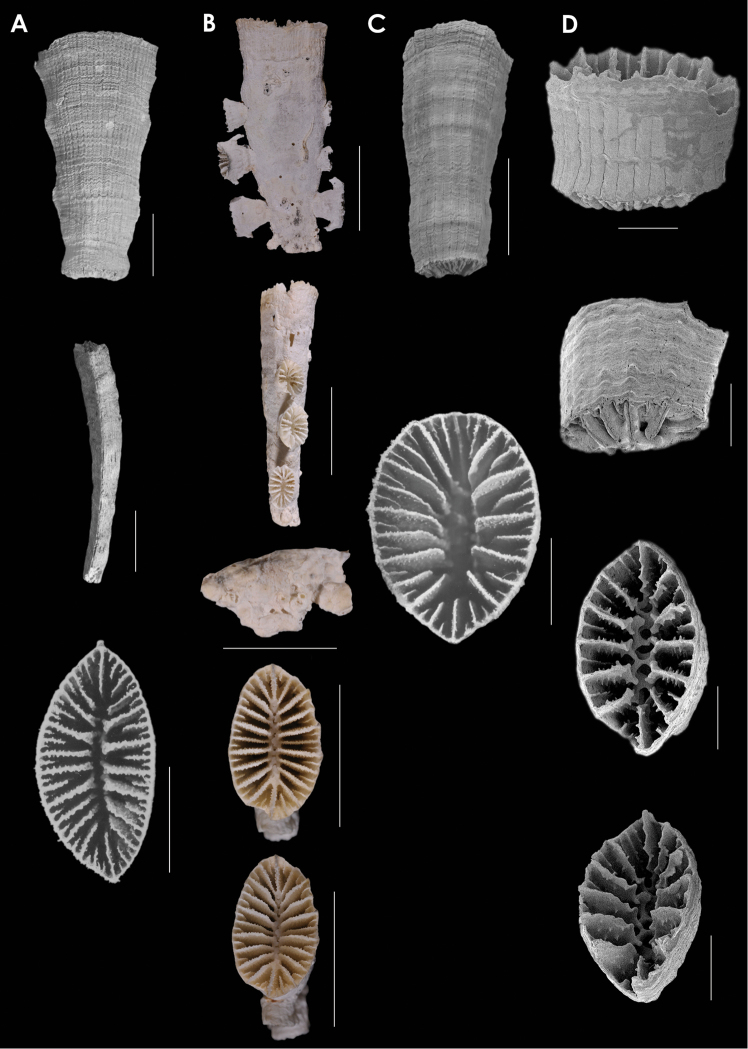
**A** *Truncatoflabellum arcuatum*, lateral and calicular views, holotype, NZOI H633, Norfolk Ridge; edge view, paratype, USNM 94280, Norfolk Ridge **B** *Blastotrochus nutrix*, USNM 97553, *Siboga*, Indonesia **C** *Placotrochides scaphula*, USNM 94273, NZOI G941, New Zealand **D** *Placotrochides minuta*, holotype, Australian Museum G16747, Flores Sea. Scale bars: 10 mm (**A–C**), except for calice of **A** and basal scar of **B**, which are 5 mm; 1 mm (**D**).

### Truncatoflabellum phoenix

Taxon classificationAnimaliaScleractiniaFlabellidae

Cairns, 1995

Fig. 2A

Truncatoflabellum sp. B. Cairns, 1994: 75, 79, pl. 33i, l.
Truncatoflabellum phoenix Cairns, 1995: 115–116, pl. 37i, 38a-f.—Cairns and Zibrowius 1997: 171.—Cairns 1999: 121.—Ogawa 2006: 16, pl. 2, fig. 5a-b.

#### New records.

USGS 25734, Vanuatu, Espiritu Santo Island, Late Pleistocene, 1 specimen, USNM 100175; Ryukyu Islands, Okinawa, Horseshoe Cliffs, 1 km NNW Onna Village (26°30'N, 127°50’54"E), 67-79 m, 6 specimens, USNM 87712, 88380, 88382, 88383, and 100674.

#### Distribution.

Late Pleistocene: Vanuatu. Holocene: southern Japan, Philippines, Indonesia, Wallis and Futuna, New Caledonia, Kermadec Islands, 18-441 m.

#### Remarks.

This is the smallest of the *Truncatoflabellum* species, having a GCD rarely more than 5 mm, but capable of multiple apical regeneration (Fig. 2A, top) resulting in coralla as long as 17.5 mm.

### Truncatoflabellum gippslandicum

Taxon classificationAnimaliaScleractiniaFlabellidae

(Dennant, 1899)

Fig. 2B

Flabellum gippslandicum Dennant, 1899: 112–113, pl. 2, figs 1a–b.—Felix 1927: 409.—Bell 1981: 11 (type deposition).—Fitzgerald and Schmidt: 3 (color fig).
Truncatoflabellum gippslandicus (*sic*): Cairns 1989b: 61.

#### Distribution.

Miocene: Gippsland Lake area of Victoria, Australia; Middle Miocene of Beaumaris, Victoria.

#### Remarks.

The two syntypes of *Truncatoflabellum gippslandicum* were reported by Bell (1981) from the NMV (P27064). No other records of this species are known, and the information presented in the key and comparative Table 2 is taken from the original description.

**Table 2.** Tabular key to all species of *Truncatoflabellum* (pr = pair, NC = New Caledonia; NZ = New Zealand; IWP = Indo-West Pacific)

	Thecal Edge Ornamentation	Edge angle; Face angle	GCD:LCD	H:GCD	GCD max.	Color	GSD:GCD; GSD max.	Septal symmetry (max number of septa)	Upper outer septal margin notched	Unique features	Distribution; depth
***phoenix***	1–2+ pr spines	0–10°; 0°	1.3–2.3	up to 4.3	5.9 mm	Lt. brown	0.86–1.0; 4.3 mm	S1-2>S3>S4 (24–32)	No	Corallum often elongated by rejuvenescence	Japan to Kermadecs; 18–441 m
***gippslandicum***	1 basal pr spines	0–10°; 10°	2.3	1.9	16 mm	Fossil	0.71; 10 mm	S1-3>S4>S5 (76)	No		Miocene: Victoria
***victoriae***	1 basal pr spines	15–20°; 11–16°	1.4	1.3	11.8 mm	Fossil	0.64; 7.6 mm	S1-2>S3>S4 (32–40)	No		Oligocene to M. Miocene: Victoria
***dens***	Small crests	Bimodal; 14–18°	1.7–2.3	1.5–1.7	13.8 mm	Red-brown	0.18–0.19; 1.6 mm	S1°>S2°>S3° (56)	No	Anthocaulus often remains attached	Philippines to NZ; 286–555 m
***zuluense***	0–1 basal pr spines	28–38°; 18–22°	1.4–1.8	0.8	13.2 mm	Striped	0.52; 6.5 mm	S1-2>S3>>S4> S5 (56)	No	Anthocaulus often remains attached	South Africa; 62–84 m
***pusillum***	2–4 pr spines	14–18°; 18–20°	1.4–1.7	1.5–1.6	8.4 mm	Striped	0.41–0.48; 3.2 mm	S1-2>S3>S4 (32–48)	No		IWP; 85–460 m
***angustum***	3–4 pr spines	28–52°; 17–22°	1.8–2.3	1.2–1.7	14 mm	Red-brown basally	0.3; 2.5 mm	S1-2>S3>S4>S5 (56)	Yes		Philippines to Queensland; 195–530 m
***angiostomum***	1 pr basal spines	105–200°; 15–25°	2.9–3.2	0.67–0.81	63 mm	White	0.08–0.09; 4.3 mm	S1-4>S5>S6>S7 (268)	Yes	Calice arched	North and west Australia; 15–176 m
***macroeschara***	1 pr basal spines	55–87°; 22–27°	2.5–3.1	0.64–1.0	46 mm	White	0.35–0.53; 30.4 mm	S1-4>S5>S6>S7 (192)	No		Australia; 18–201 m
***veroni***	1 pr basal spines	94–127°; 23–32°	3.0–4.8	0.5–0.56	57 mm	White	0.33; 27 mm	S1-4>S5>S6>S7 (192–212)	Yes		Australia; 15–176 m
***gambierense***	1 pr spines	30–38°; 15–20°	1.6–3.2	1.4–1.8	14.5 mm	Fossil	0.52–0.67; 7.2 mm	S1-2>S3>S4?S5 (56)	No	Anthocaulus slender, remains attached	Middle Miocene; Victoria
***incrustatum***	1 pr basal spines	23–32°; 15–19°	1.6–2.1	1.2–1.5	28 mm	Blackish	0.24–0.38; 10 mm	S1-2>S3>S4>S5 (96)	No		Japan to Philippines; 30–315 m
***sphenodeum***	1 basal pr spines	32°; 18°	1.67	1.33	15 mm	Fossil	0.25–0.33; 3.5 mm	S1-3>S4>S5 (60–75)	No		M. Eocene to M. Miocene: NZ
***irregulare***	1 pr basal spines	36–43°; 19°	1.6–2.0	1.4	28 mm	White	0.32–0.5; 4 mm	S1°>S2°>S3° (72–80)	Yes		Japan to Philippines; 11–55 m
***crassum***	1 pr basal spines	40–50°; 18–28°	1.3–1.8	0.75–0.85	29 mm	White	0.21–0.29; 6.3 mm	S1-2>S3>S4> S5>S6 (114)	Yes		IWP: 31–430 m
***aculeatum***	1 pr basal spines	31–82°;17–31°	1.8–3.7	0.56–0.71	41 mm	Milky white	0.35–0.44; 15 mm	S1°>S2°>S3° (50–72)	Yes		Japan to w. Australia; 11–132 m
***mortenseni***	1 pr spines	49–61°; 23–31°	1.65–1.85	0.75–0.81	23 mm	Striped	0.32–0.40; 7 mm	S1-3>S4>S5 (96)	Yes	Anthocyathus often remains attached	Philippines to New Caledonia; 50–455 m
***australiensis***	2–3 pr spines	44–73°; 18–28°	1.9–2.4	0.64–0.83	25 mm	Striped	0.36–0.48; 8.6 mm	S1-3>S4>S5 (96)	No		W. Australia; 90–220 m
***candeanum***	3 long pr spines	40–80°; 30–41°	1.6–2.0	0.73–0.76	32 mm	Striped	0.26–0.29; 5.7 mm	S1°>S2°>S3° (72–96)	No		Japan to Philippines, NC; 70–290 m
***compressum***	2–3 pr spines	53–67°; 24–29°	1.9–3.1	0.6–0.8	40 mm	White	0.37–0.43; 13.7 mm	S1-4>S5>S6 (192)	Yes		Philippines to Indian Ocean; 12–256 m
***martensii***	3 pr spines	40–105°; 14–19°	2.0–2.4	0.83–1.0	29 mm	Red or brown	0.28–0.30; 9.3 mm	S1-3>S4>S5>S6 (126)	No	Thecal edges acute	New Caledonia to Andaman Sea; 139–275 m
***mozambiquensis***	1–2 pr spines	39–60°; 22–32°	1.4–2.2	0.97–1.4	27 mm	Blackish	0.19–0.26; 6.9 mm	S1-2>S4>S5 (96)	No	C1–3 ribbed	Mozambique; 106–112 m
***vigintifarium***	2–3 pr spines	67–84°; 25–30°	1.95–2.40	0.84–0.91	27 mm	Striped	0.13; 3.6 mm	S1°>S2°>S3° (80)	No		New Caledonia, Queensland; 179–1050 m
***spheniscus***	1 basal pr spines	65–118°; 16–31°	2.8–4.1	0.76–0.81	50 mm	Striped	0.22–0.49; 18 mm	1°>2°>3°>4° (190)	Yes	Calice arched	Japan to Australia; 2–174 m
***cumingi***	2–3 pr spines	31–44°; 18–236	1.8–2.0	1.0–1.13	20 mm	White	0.37–0.41; 9 mm	S1°>S2°>S3° (72)	No		Philippines to W. Australia; 46–132 m
***vanuatu***	4–5 pr spines	20–27°; 12–17°	1.6–1.8	1.7–1.9	26 mm	White	0.22–0.29; 4.9 mm	S1°>S2°>S3° (80)	No	Axial septal edges straight	Vanuatu, NC; 240–335 m
***duncani***	5 pr spines	54–72°; 27°	1.4–1.7	0.93–1.04	31 mm	Fossil	0.27–0.29; 10.5 mm	S1-3>S4>S5>S6 (104)	Attenuate		L. Oligocene-M. Miocene: Victoria
***multispinosum***	5–7 pr spines	41–56°; 19–32°	1.7–2.1	0.93–1.02	32 mm	Brown	0.23–0.30; 7.3 mm	S1-3>S4>S5>S6 (100)	No		W. Indian Ocean, NC; 62–183 m
***paripavoninum***	None, thecal edges acute	65–138°; 32–62°	1.4–2.0	0.77–1.0	62 mm	Lt brown	0.17–0.34; 14.5 mm	S1-3>S4>S5>S6 (192)	No		Philippines to Laccadive Sea; 394–1450 m
***stabile***	None, thecal edges rounded	59–90°; 40–60°	1.4–1.7	1.0–1.15	52 mm	White	0.14–0.28; 7 mm	S1-3>S4>S5>S6 (104)	No	Costae ribbed	Japan, Mozambique, Cape Verde; 786–3010 m
***inconstans***	None, thecal edges rounded	38–52°; 25°	1.5–2.5	0.10–1.5	44 mm	White	0.13–0.18; 5 mm	S1-3>S4>S5>S6 (171)	No	C1–3 ribbed	South Africa; 23–130 m
***corbicula***	None, thecal edges rounded	16–21°; 15°	1.5–2.2	0.97	19 mm	Fossil	0.64–0.67; 12 mm	S1-2>S3>S4 (48)	No		L. Oligocene, New Zealand
***truncum***	None, thecal edges rounded	45–70°; 22–38°	1.4–2.2	0.9–1.2	38 mm	White	0.25–0.27; 9.5 mm	S1-3>S4>S5 (96)	No	C1–3 ribbed	Peru to Falklands; 595–1896 m
***trapezoideum***	None, thecal edges rounded	80°; ?	1.35	0.69	28 mm	White	0.29; 8.1 mm	S1-2>S3>S4>S5 (88)	No	C1–2 ribbed	Marcus-Necker Ridge; 1630 m
***formosum***	2 pr crests	37–59°; 18–31°	1.4–1.9	1.05–1.2	27 mm	Striped	0.26; 5.5 mm	S1°>S2°>S3° (80)	Attenuate		Philippines to SW Indian Ocean; 42–933 m
***carinatum***	Disjunct crests	35–57°; 18–32°	1.6–1.9	0.88–1.2	23 mm	Red-brown	0.22–0.24; 5.2 mm	S1-3>S4>S5>S6 (104)	No		S. China Sea to Mozambique; 30–274 m
***gardineri***	Crests	21–35°; 14–18°	1.3–1.5	1.3–1.9	20 mm	White or striped	0.37–0.49; 5.3 mm	S1-2>S3>S4 (48)	No		Japan, S. Africa; 100–144 m
***arcuatum***	Low crests	14–15°; 8–11°	1.8–2.6	2.9–3.5	12 mm	White	0.50–0.55; 5.9 mm	S1-2>S3>S4>S5 (60)	No	Axial septal edges very sinuous	North of New Zealand; 350–364 m

### Truncatoflabellum victoriae

Taxon classificationAnimaliaScleractiniaFlabellidae

(Duncan, 1864)

Fig. 2C

Flabellum victoriae Duncan, 1864: 162–163, pl. 5, fig. 2a–c; 1870: 299, 312, pl. 19, fig. 11.—Tenison-Woods 1878b: 312.—Felix 1927: 415 (synonymy).
?Flabellum simplex Tenison-Woods, 1878: 13.—Squires 1958: 66 (type lost).
Truncatoflabellum victoriae : Cairns, 1989b: 61, pl. 37i.

#### New records.

Muddy Creek, near Hamilton, Victoria, Australia, Balcombian (Middle Miocene), 13 specimens, USNM 67962 and 68005; Balcombe’s Bay, Port Phillip, Victoria, Balcombian (Middle Miocene), 11 specimens, USNM 68000 and M353582; Balcombe’s Bay, Mornington, Balcombian (Middle Miocene), 3 specimens, USNM M353583; Spring Creek, Torquay, Victoria, Janjukian (Late Oligocene), 8 specimens, USNM 1283656.

#### Distribution.

Late Oligocene (Janjukian), Victoria; Middle Miocene (Balcombian), Muddy Creek, Geelong, Victoria, and Balcombe’s Bay, Victoria.

### Truncatoflabellum dens

Taxon classificationAnimaliaScleractiniaFlabellidae

(Alcock, 1902)

Fig. 2D

Flabellum dens Alcock, 1902: 32, pl. 4, figs 30, 30a.—Cairns 1989b: 54, Table 6, pl. 28g–k.
Truncatoflabellum dens : Cairns 1995: 114–115 (in part: pl. 37g).—Cairns and Zibrowius 1997: 170–171, 173.—Cairns 1999: 120, fig. 20a.

#### Distribution.

Philippines, Indonesia, New Caledonia, Vanuatu, Wallis and Futuna, New Zealand, 286–555 m.

### Truncatoflabellum zuluense

Taxon classificationAnimaliaScleractiniaFlabellidae

Cairns in Cairns & Keller, 1993

Fig. 3A

Truncatoflabellum zuluense Cairns in Cairns & Keller, 1993: 267–268, figs 11F–G.—Cairns 1995: 115 (comparison to *Truncatoflabellum dens*).

#### Distribution.

Off Natal, South Africa, 62-84 m.

### Truncatoflabellum pusillum

Taxon classificationAnimaliaScleractiniaFlabellidae

Cairns, 1989b

Fig. 3B

Truncatoflabellum pusillum Cairns, 1989b: 71_72, Table 6, pl. 37a–e.—Cairns and Keller 1993: 265, fig. 11E.—Cairns 1995: 115 (comparison to *Truncatoflabellum dens*).—Cairns and Zibrowius 1997: 170.—Cairns 1999: 120, fig. 20a

#### Distribution.

Philippines, Indonesia, Vanuatu, New Caledonia, southwest Indian Ocean off Mozambique, 85-460 m.

### Truncatoflabellum angustum

Taxon classificationAnimaliaScleractiniaFlabellidae

Cairns & Zibrowius, 1997

Fig. 3C

Truncatoflabellum dens : Cairns 1995: in part (pl. 37f, h).
Truncatoflabellum angustum Cairns & Zibrowius, 1997: 172–173, figs 23c–f.—Cairns 1999: 121, fig. 20b.—Cairns 2004: 308.

#### Distribution.

Philippines, Indonesia, Vanuatu, Wallis and Futuna, Kermadec Islands, off Queensland, 195–530 m.

### Truncatoflabellum angiostomum

Taxon classificationAnimaliaScleractiniaFlabellidae

(Folkeson, 1919)

Fig. 3D

Flabellum angiostomum Folkeson, 1919: 5, pl. 1, figs 1-3.
Truncatoflabellum angiostomum : Cairns 1998: 395–396, Table 4, figs 7a–c, 8a; 2004: 308 (synonymy).

#### New records.

SIPHILEXP M-21, 8°45'S, 145°05'08"E (off mouth of Fly River, Bramble Island, Papua New Guinea), 55 m, USNM 1130683; Karubar 65, 9°14'01"S, 132°28'28"E, 174-176 m, 1, USNM 97256.

#### Distribution.

Western and Northern Australia, Papua New Guinea, 15-176 m.

### Truncatoflabellum macroeschara

Taxon classificationAnimaliaScleractiniaFlabellidae

Cairns, 1998

Fig. 4A

Truncatoflabellum macroeschara Cairns, 1998: 401, Table 4, figs 8d–e, g–1; 2004: 309 (synonymy).—Kitahara et al. 2010: fig. 1.

#### Distribution.

Off Western Australia and Queensland, 18-201 m.

#### Remarks.

*Truncatoflabellum macroeschara* belongs to a group of three western Australian species that have very large coralla, often including some S7, the other two species being *Truncatoflabellum veroni* and *Truncatoflabellum angiostomum*. It differs from those two species as well as all others in the genus by having a very large scar diameter.

### Truncatoflabellum veroni

Taxon classificationAnimaliaScleractiniaFlabellidae

Cairns, 1998

Fig. 4B

Truncatoflabellum spheniscus : Cairns and Zibrowius: 165–166 (in part: USNM 93197 and USNM 97499).
Truncatoflabellum veroni Cairns, 1998: 400, Figs 7g–i, 8c; 2004: 309 (synonymy).

#### Distribution.

Off Western and Northern Australia, off Queensland, 15–176 m.

### Truncatoflabellum gambierense

Taxon classificationAnimaliaScleractiniaFlabellidae

(Duncan, 1864) comb. n.

Fig. 4C

Flabellum gambierense Duncan, 1864: 163, pl. 5, fig. 3a-c; 1870: 299–300, 308, 310, 312, pl. 19, figs 9–10.—Tenison-Woods 1878b: 312.—Felix 1927: 409.—Fitzgerald and Schmidt: 3 (color figure).

#### New records.

Spined coralla: USGS 10809, Balcombe’s Bay, Mornington, Victoria (Balcombian, Middle Miocene), 2 specimens, USNM 1295473. Non-spined coralla: Muddy Creek, Victoria (Balcombian, Middle Miocene), 9 specimens, USNM 67958, 353989, and M353589; Balcombe’s Bay, Mornington, Victoria (Balcombian, Middle Miocene), 6 specimens, USNM M353581 and M353580.

#### Distribution.

Middle Miocene: Mount Gambier, S. Australia; Cape Otway, Balcombe’s Bay, Mornington, and Beaumaris, Victoria.

#### Remarks.

In the original description, Duncan (1864) described the species as not having thecal edge spines, but in 1870 said that the coral has “often small spines nearer the calice than the pedicel.” Indeed, some specimens of this distinctively-shaped species have spines (traditional *Truncatoflabellum*) and others do not (see New Records). Ordinarily, if a species of *Truncatoflabellum* bears thecal edge spines then all specimens of that species will bear spines. Thus, this variation in character is unusual and may be indicative of the early evolution in the genus when spination and transverse division were still experimental, as *Truncatoflabellum gambierense* is one of those species that shows a crescentric transverse weakness in its corallum but the anthocyathus usually remains attached to the anthocaulus, possibly the ancestral condition for the species.

### Truncatoflabellum irregulare

Taxon classificationAnimaliaScleractiniaFlabellidae

(Semper, 1872)

Fig. 4D

Flabellum irregulare Semper, 1872: 242–245, figs 1–3, pl. 16, figs 7–17.
Not Flabellum irregulare Tenison-Woods, 1878b: 313, pl. 4, Fig. 2 (junior homonym, = *Truncatoflabellum cumingi*).
Truncatoflabellum irregulare : Cairns 1989b: 67–68, Table 6, pls. 34i–k, 35a–c (synonymy). —Cairns and Zibrowius 1997: 168.

#### New record.

Ryukyu Islands, Horseshoe Cliffs (26°30'00"N, 127°50'54"E), 55 m, 1 specimen, USNM 87710.

#### Distribution.

Philippines, Indonesia, Ryukyu Islands, 11-55 m.

### Truncatoflabellum incrustatum

Taxon classificationAnimaliaScleractiniaFlabellidae

Cairns, 1989b

Fig. 5A

Flabellum irregulare : Gerth 1921: 402, pl. 57, fig. 15.—Cairns 1989b: 63, Table 6, pl. 37f.
Truncatoflabellum incrustatum Cairns, 1989b: 68–69, Table 6, pl. 35d–e.—Cairns and Zibrowius 1997: 168.

#### New record.

*Tansei Maru* KT9202-YT1, 30°14'48"N, 130°46'06"E, 80–88 m, 5 specimens, USNM 92788.

#### Distribution.

Lower Miocene of Java (Gerth, 1921). Holocene: Philippines; Indonesia; Ryukyu Islands, Japan, 30-315 m.

### Truncatoflabellum sphenodeum

Taxon classificationAnimaliaScleractiniaFlabellidae

(Tenison-Woods, 1880) comb. n.

Fig. 5B

Flabellum sphenodeum Tenison-Woods, 1880: 14, figs 12a-c.—?Hayward, 1977: 105-106, fig. 8.
?Flabellum attenuatum Tenison-Woods, 1880: 15, fig. 15.
Flabellum rubrum sphenodeum : Squires 1958: 66–67, pl. 11, figs 21–25 (lectotype designated).
?Flabellum sp. A Hayward, 1977: 106, fig. 9.

#### New record.

Junction of Porter and Thomas Rivers, New Zealand, S66/74, NZGS 3350, Duntroonian (early Oligocene), 3 specimens, USNM 67908.

#### Distribution.

Middle Eocene (Bortonian) to Middle Miocene (Waiauan) of New Zealand (Squires 1958).

#### Remarks.

According to the records of Squires (1958), this would be the oldest *Truncatoflabellum*, being reported from the Bortonian (Middle Eocene) of New Zealand.

The specimens reported by Hayward (1977) as “*Flabellum*” *sphenodeum* and “*Flabellum*” sp. A have much larger basal scars and shorter coralla than typical *Flabellum sphenodeum* and are thus not included with this species. Specimens in the NMNH that may be the same are USNM 67932 and 67928, and may represent an undescribed species.

### Truncatoflabellum crassum

Taxon classificationAnimaliaScleractiniaFlabellidae

(Milne Edwards & Haime, 1848)

Fig. 5C

Flabellum crassum Milne Edwards & Haime, 1848: 276–277, pl. 8, figs 8, 8a.
Flabellum stokesi : Scheer and Pillai 1974: 62–63, pl. 29, figs 1–2.
Truncatoflabellum crassum : Cairns 1989b: 64–65, Table 6, pl. 32d–f (synonymy).

#### New records.

*Albatross* 5091, 35°04'10"N, 139°38'12"E, 366 m, 1 specimen, USNM 92812; *Albatross* 5270, 13°35'45"N, 120°58'30"E, 430 m, 1 specimen, USNM 1130686; *Anton Bruun* 1–38, 14°07'N, 95°05'E, 69–73 m, 1 specimen, USNM 1015342; *Anton Bruun* 260A, 26°15'N, 56°46'E, 91 m, 1 specimen, USNM 1015348; *Anton Bruun* 9–447, 10°00'N, 51°15'E, 59–61 m, 1 specimen, USNM 1015346; *Anton Bruun* 9–451, 11°04'N, 51°15'E, 76–80 m, 14 specimens, USNM 98977; *Anton Bruun* 9–453, 11°11'N, 51°14'E, 47–49 m, approx.. 200 specimens, USNM 77040; *Anton Bruun* 9–456, 11°14'N, 51°08'E, 27–31 m, 1 specimen, USNM 1015285; 11°15'N, 51°12'E, 50 m, 10 specimens, USNM 1015170.

#### Distribution.

Philippines, Sagami Bay (Japan), Gulf of Aden, Persian Gulf, Great Nicobar, Andaman Islands, 31–430 m.

### Truncatoflabellum aculeatum

Taxon classificationAnimaliaScleractiniaFlabellidae

(Milne Edwards & Haime, 1848)

Fig. 5D

Flabellum aculeatum Milne Edwards & Haime, 1848: 272, pl. 8, figs 3, 3a.—Cairns 1989a: 643, fig. 1 (lower).
Flabellum spinosum Milne Edwards & Haime, 1848: 271, pl. 8, fig. 4.
Flabellum variable Semper, 1872: 245–251, pl. 17, pl. 18, figs 1–10.
Flabellum rubrum : Umbgrove 1938: 264 (in part).
Truncatoflabellum aculeatum : Cairns 1989b: 61–64, Table 6, pl. 31h-l, 32a-c.—Cairns and Zibrowius 1997: 166–167.—Cairns 1998: 399–400, Table 4; 1999: 123; 2004: 308 (synonymy).

#### New records.

*Tansei Maru* KT9202, YT1, 30°14'48"N, 130°46'06"E, 80–88 m, 2 specimens, USNM 92790; Singapore, depth unknown, 1 specimen, USNM 1279597.

#### Distribution.

Pleistocene: Indonesia. Holocene: Okinawa, Philippines, Indonesia, Vanuatu, off Queensland, Northern Territory and Western Australia, 11–132 m.

### Truncatoflabellum mortenseni

Taxon classificationAnimaliaScleractiniaFlabellidae

Cairns & Zibrowius, 1997

Fig. 6A

Truncatoflabellum mortenseni Cairns & Zibrowius, 1997: 171–172, figs 22g-h.—Cairns 1999: 122–123.

#### Distribution.

Philippines, Indonesia, Vanuatu, Wallis and Futuna, New Caledonia, 50–455 m.

### Truncatoflabellum australiensis

Taxon classificationAnimaliaScleractiniaFlabellidae

Cairns, 1998

Fig. 6B

Truncatoflabellum australiensis Cairns, 1998: 396–399, Table 4, figs 7d-f, 8b; 2004: 308.—Kitahara et al. 2010: fig. 1.

#### Distribution.

Western Australia, 90–220 m.

### Truncatoflabellum candeanum

Taxon classificationAnimaliaScleractiniaFlabellidae

(Milne Edwards & Haime, 1848)

Fig. 6C

Flabellum candeanum Milne Edwards & Haime, 1848: 278, pl. 8, fig. 13.—Not Duncan, 1864: 163 (=*Truncatoflabellum duncani*, herein).
Flabellum elegans Milne Edwards & Haime, 1848: 277.
Truncatoflabellum candeanum : Cairns 1989b: 70–71, Table 6, pl. 36d-h (synonymy, neotype designated); 1994: 76–77, pl. 33e-f.—Cairns and Zibrowius 1997: 167.—Cairns 1999: 123–124.—Kitahara et al. 2010: fig. 1.

#### Distribution.

Southern Japan, Philippines, Indonesia, Vanuatu, New Caledonia, 70–290 m.

### Truncatoflabellum compressum

Taxon classificationAnimaliaScleractiniaFlabellidae

(Lamarck, 1816)

Fig. 6D

Fungia compressa Lamarck, 1816: 235; 1827: pl. 483, fig. 2.
Flabellum compressum : Milne Edwards & Haime, 1848: 273–274 (synonymy).—Duncan 1864: 167.
Flabellum stokesii Milne Edwards & Haime, 1848: 278, pl. 8, fig. 12.—Moseley 1881: 172–173 (in part: *Challenger* 190).—Gerth 1921: 402, pl. 567, fig. 14.—Not Umbgrove 1950: 640.—Not Scheer and Pillai 1974: 62 (=*Truncatoflabellum crassum*).
Flabellum oweni Milne Edwards & Haime, 1848: 279, pl. 8, fig. 9.
Truncatoflabellum stokesi : Cairns 1989b: 66, Table 6, pl. 33b-h, j (synonymy).
Truncatoflabellum compressum : Cairns 1989b: 61 (listed).

#### Distribution.

Miocene: Java. Holocene: Philippines, Indonesia, “Indian Ocean” (Lamarck, 1816), 12–256 m.

#### Remarks.

This species, with a name overlooked since 1864, was beautifully illustrated by Lamarck (1827). Its description and illustration (Fig. 6D, top) leave little doubt that it is the species that has become known as *Truncatoflabellum stokesi*.

### Truncatoflabellum martensii

Taxon classificationAnimaliaScleractiniaFlabellidae

(Studer, 1878)

Fig. 7A

Flabellum martensii Studer, 1878: 630–631, pl. 1, figs 4a-b.
Flabellum paripavoninum : Wells 1984: 214–215, fig. 4, 6–7.
Truncatoflabellum martensii : Cairns 1989b: 61, Table 6, pl. 37, figs g–h; 1999: 124, figs 21a–f (synonymy); 2004: 309.
Truncatoflabellum sp. Cairns & Kitahara, 2012, pl. 23, figs C–F.

#### New records.

*Anton Bruun* 1–22A, 10°39'N, 97°06'E, 275 m, 5 specimens, USNM 1015345; *Anton Bruun* 4B-230B, 23°31'N, 66°55'E, 88 m, 1 specimen, USNM 1015347.

#### Distribution.

Late Pleistocene: Vanuatu (Wells, 1984). Holocene: New Caledonia, Vanuatu, off Brisbane, Andaman Sea, 139–275 m.

### Truncatoflabellum mozambiquensis sp. n.

Taxon classificationAnimaliaScleractiniaFlabellidae

http://zoobank.org/5F659B28-7F5B-45D0-9E87-0CD7D9DC7731

Fig. 7B

#### Types.

Holotype: *Anton Bruun* 7–372L, 25°07'S, 34°34'E, 112 m, grey sandy mud, USNM 91764. Paratypes: *Anton Bruun* 7–372L, 232 coralla, USNM 1283832; *Anton Bruun* 7–371F, 24°46'S, 35°18'E, 110 m, 1 specimen, USNM 91762; *Anton Bruun* 7–372J, 25°07'S, 34°34'E, 106 m, 28 specimens, USNM 91763.

#### Description.

The anthocyathus has straight, rounded thecal edges, having an edge angle of 39–60°; the face angle ranges from 22–28°. The largest specimen has a GCD of 26.5 mm, whereas the holotype measures 23.4 × 11.2 in calicular diameter, 24.5 mm in height, and 5.3 mm in greater scar diameter. The GCD:LCD ratio is 1.4–2.2; the H:GCD is 1.0–1.4; the GSC:GCD is 0.19–0.26, with the GSD up to 6.9 mm in length. One pair of very short (rarely more than 1 mm long) and often broken and worn thecal edges spines occur near the basal scar; another pair often is present more distally. The thecal faces bear low ribbing corresponding to the C1–3. The corallum, although worn, sometimes has a blackish color. The septa are arranged in five cycles: S1–3>S4>S5, mature coralla having 96 septa. The lower axial septal edges are highly sinuous, and merge into a rudimentary elongate columella. The upper outer septal edges are not notched. The fossa is deep and narrow, although almost all coralla examined were partially damaged, making observations of the septa and fossa tentative.

Anthocauli are rare, only four of the 262 (1.5%) specimens representing this juvenile stage. It is small, only about 4.1 mm in height with a circular attached pedicel 2 mm in diameter, and a distal calice 5–6 mm in greater diameter corresponding to the scar diameter of the anthocyathus. It has three cycles of septa.

#### Distribution.

Off southern Mozambique, 106–112 m.

#### Remarks.

As suggested by the key, *Truncatoflabellum mozambiquensis* is most similar to *Truncatoflabellum martensii*, but can be distinguished by its smaller basal scar, higher H:GCD ratio, rounded thecal edges, and tendency to have one (or occasionally two) pairs of thecal edge spines vs. three pairs for *Truncatoflabellum martensii* (Table 2).

#### Etymology.

Named for the country from which it was found.

### Truncatoflabellum vigintifarium

Taxon classificationAnimaliaScleractiniaFlabellidae

Cairns, 1999

Fig. 7C

Truncatoflabellum vigintifarium Cairns, 1999: 121–122, figs 2c–f; 2004, 309.

#### Distribution.

Vanuatu, New Caledonia, off Queensland, 179–1050 m.

### Truncatoflabellum spheniscus

Taxon classificationAnimaliaScleractiniaFlabellidae

(Dana, 1846)

Fig. 7D

Euphyllia spheniscus Dana, 1846: 160–161, pl. 6, figs 1a–e.
Flabellum sumatrense Milne Edwards & Haime, 1848: 271.
Flabellum debile Milne Edwards & Haime, 1848: 274, pl. 8, fig. 2.
Flabellum affine Milne Edwards & Haime, 1848: 274, pl. 8, fig. 10.
Flabellum bairdi Milne Edwards & Haime, 1848: 274–275.
Flabellum profundum Milne Edwards & Haime, 1848: 276.
Flabellum elongatum Milne Edwards & Haime, 1848: 275, pl. 8, fig. 7.
Flabellum crenulatum Milne Edwards & Haime, 1848: 277.
Flabellum variabile : Gerth, 1921: 401, pl. 57, fig. 30.—Cairns 1989b: Table 6, pl. 33, pl. 33a.
Flabellum rubrum debile : Yabe & Eguchi, 1941: 269, figs 5–6.
Truncatoflabellum bairdi : Cairns 1989b: 66–67, Table 6, pl. 33k, 34a–c.
Truncatoflabellum profundum : Cairns 1989b: 67, Table 6, pl. 34d–h.
Truncatoflabellum spheniscus : Cairns 1989b: 65–66, pl. 32g-k (synonymy); 1994: 76, pl. 33a–d (synonymy); 1999: 399, Table 4; 2004: 309.

#### New records.

*Albatross* 5483, 10°27'30"N, 125°19'15"E, 135 m, 4 specimens, USNM 1130688; *Albatross* 5593, 4°02'20"N, 118°11'20"E, 69 m, 1 specimen, USNM 1130687.

#### Distribution.

Pliocene: Java (Gerth 1921; Yabe and Eguchi 1941). Holocene: Japan, Indonesia, circum-Australia, 2–174 m.

#### Remarks.

The name *spheniscus*, Latin for small wedge, is treated as a noun in apposition and thus does not match gender with the genus.

### Truncatoflabellum cumingi

Taxon classificationAnimaliaScleractiniaFlabellidae

(Milne Edwards & Haime, 1848)

Fig. 8A

Flabellum cumingii Milne Edwards & Haime, 1848: 275, pl. 8, fig. 11.
Flabellum irregulare Tenison-Woods, 1878b: 313 (junior homonym of *Flabellum irregular* Semper, 1872).
Truncatoflabellum cumingi : Cairns 1989b: 69, Table 6, pl. 35f–i (neotype designated, synonymy); 2004: 309 (synonymy).

#### Distribution.

Philippines, Indonesia, off New South Wales and Western Australia, 46–132 m.

### Truncatoflabellum vanuatu

Taxon classificationAnimaliaScleractiniaFlabellidae

(Wells, 1984)

Fig. 8B

Flabellum vanuatu Wells, 1984: 215, figs 4 (11–12), 5 (1).
Truncatoflabellum vanuatu : Cairns 1989b, Table 6, 69, pl. 36c; 1999: 123.
Truncatoflabellum sp. A: Kitahara et al. 2010: fig. 1.
Truncatoflabellum sp. B: Kitahara et al. 2010: fig. 1.

#### New records.

Kere River, Espiritu Santo, Vanuatu, Late Pleistocene, USGS 25715, 25717, and 27718, 35 specimens, USNM 100195, 99485, and 73972, respectively.

#### Distribution.

Late Pleistocene: Vanuatu. Holocene: Vanuatu, Wallis and Futuna, New Caledonia, 240–335 m.

### Truncatoflabellum duncani sp. n.

Taxon classificationAnimaliaScleractiniaFlabellidae

http://zoobank.org/67F30A3A-308C-46E9-8A1C-755DA0D9920B

Fig. 8C

Flabellum candeanum : Duncan 1864: 163; 1870: 300, pl. 20, fig. 1.
Truncatoflabellum candeanum : Cairns 1989b: 61, pl. 36i-j.

#### Types.

Holotype: USGS 10809, Mornington, Balcombe’s Bay, Victoria, Balcombian (Middle Miocene), USNM M353592. Paratypes: Muddy Creek, Victoria, Balcombian (Middle Miocene), 3 specimens, USNM 67959; Torquay, Balcombe’s Bay, Victoria, Janjukian (Late Oligocene), 1 specimen, USNM 1295618; 3 miles (=4.8 km) west of river Gellibrand, Otway’s region, Victoria, “Murray Tertiaries” (probably Middle Miocene) (specimen reported by Duncan, 1864, 1870), BM.

#### Description.

The anthocyathus has straight rounded thecal edges, with an edge angle of 54–72° and face angle of about 27°. The holotype is 30.8 × 18.1 mm in calicular diameter and 28.5 mm in height, with a greater scar diameter of 8.7 mm, similar in size to the specimen reported by Duncan. The GCD:LCD ratio is 1.5–2.1; the H:GCD = 0.95–1.05; and the GSD:GCD is about 0.27, with the scar reaching as long as 12 mm. Four or five pairs of prominent flattened thecal edge spines are present. The septa are quite regularly arranged in five cycles (S1–3>S4>S5), with one pair of S6 in each of the four end half-systems, resulting in 104 septa. The lower axial edges of the larger septa are only slightly sinuous, whereas the upper outer edges are gracefully attenuate, meeting the upper theca as low lamellae. The fossa is open, bordered by the axial edges of the wide S1–3. The anthocaulus is unknown.

#### Distribution.

Late Oligocene to Middle Miocene, Victoria.

#### Remarks.

As suggested by the key, *Truncatoflabellum duncani* is remarkably similar to *Truncatoflabellum multispinosum*, but can be distinguished by its attenuated upper septal margins. It is also known only from the Oligocene to Miocene of Australia, whereas *Truncatoflabellum multispinosum* is restricted to the Holocene and Late Pleistocene.

#### Etymology.

Named in honor Peter M. Duncan, who first discovered specimens belonging to this species.

### Truncatoflabellum multispinosum

Taxon classificationAnimaliaScleractiniaFlabellidae

Cairns in Cairns & Keller, 1993

Fig. 8D

**Figure 12.**
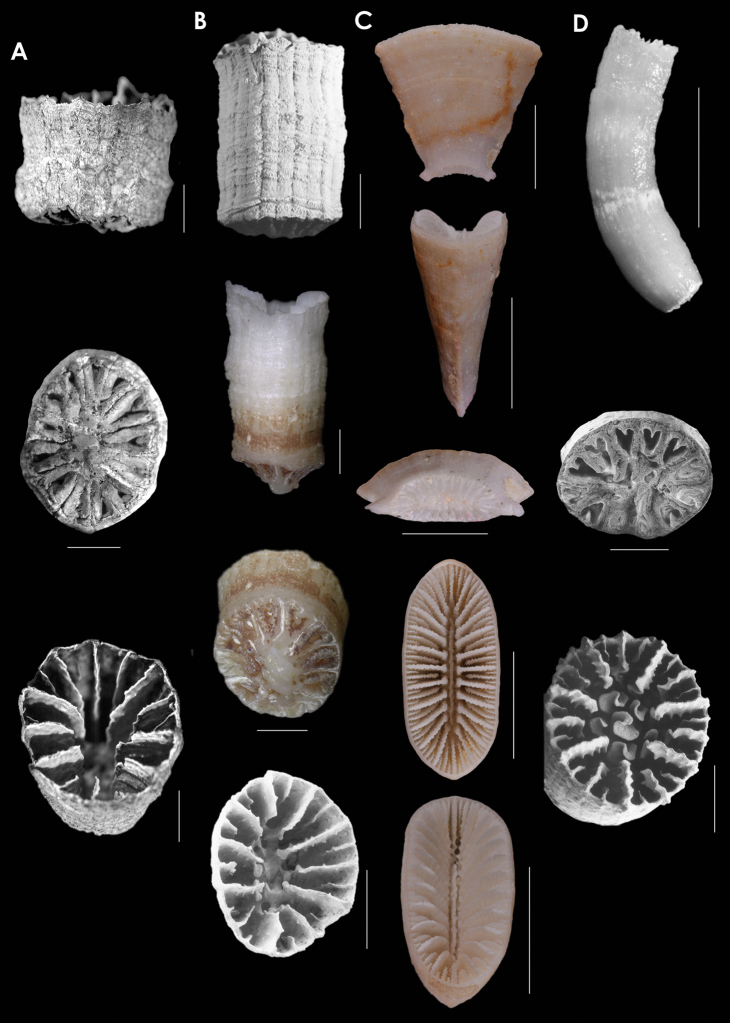
**A** *Placotrochides cylindrica*, holotype, Museum of Tropical Queensland G55627, off Queensland **B** *Placotrochides frustum*, holotype, USNM 36451, Lesser Antilles; paratype, NMC, *Hudson* 4B, Lesser Antilles **C** *Placotrochus laevis*, USNM 81994, Great Barrier Reef, Australia **D** *Falcatoflabellum raoulensis*, upper image, holotype, Museum of New Zealand, CO 258, Kermadec Ridge; lower images, paratype, USNM 94313, Kermadec Ridge. Scale bars: 1mm (**A**); 2 mm (**B**); 10 mm (**C**), except for basal scar, which is 5 mm; 1 mm (**D**), except latera view, which is 5 mm.

Truncatoflabellum multispinosum Cairns in Cairns & Keller, 1993: 268, 272, figs 11H, 12A–C.

#### New record.

USGS 25718, Kere River, Espiritu Santo, Vanuatu, Late Pleistocene, 2 specimens, USNM 100183.

#### Distribution.

Late Pleistocene: Vanuatu. Holocene: western Indian Ocean from South Africa to Tanzania, New Caledonia, 62-183 m.

### Truncatoflabellum paripavoninum

Taxon classificationAnimaliaScleractiniaFlabellidae

(Alcock, 1894)

Fig. 9A

Flabellum pari-pavoninum Alcock, 1894: 187.
Flabellum paripavoninum : Alcock 1898: 21, pl. 2, fig. 3a–b.
Truncatoflabellum paripavoninum : Cairns 1989b: 72–73, Table 6, pls. 37j–l, 38a (synonymy); 1995: 113–114, pl. 37d–e.—Cairns and Zibrowius 1997: 169, fig. 22f.—Cairns 1998: 399; 2004: 309.

#### Distribution.

Philippines, Indonesia, New Caledonia, Kermadec Islands, Western Australia, Laccadive Sea, 394–1450 m.

#### Remarks.

*Truncatoflabellum paripavoninum* belongs to a group of six species that lack thecal edge spines and crests (see Key: couplets 28–32). Except for *Truncatoflabellum inconstans*, known only from limited material from 23–130 m, these species have the greatest depth ranges of all the species in the genus often occurring deeper than 1000 m, suggesting that spines are less necessary for life at great depths. This begs the question of the function of the thecal edge spines. Even the relatively shallow species that have edge spines live at hundreds of meters of depth, far below the level at which surface turbulence would affect them. Thus the function of the thecal spines still remains unresolved.

### Truncatoflabellum stabile

Taxon classificationAnimaliaScleractiniaFlabellidae

(Marenzeller, 1904)

Fig. 9B

Flabellum stabile Marenzeller, 1904: 273-274, pl. 17, figs 12a–b.—Zibrowius 1980: 150 (types lost).
Truncatoflabellum stabile : Cairns 1989b: 61.—Zibrowius and Gili 1990: 39.—Cairns 1999: 119, figs 19i-j (synonymy).
Truncatoflabellum sp. cf. *Truncatoflabellum stabile*: Cairns and Keller 1993: 264-265, figs 10C, F.
Truncatoflabellum sp. A Cairns, 1994: 75, 79, pl. 34c–e.

#### Distribution.

Ryukyu Islands, Vanuatu, off Mozambique, Cape Verde, Madeira, 786–3010 m.

#### Remarks.

This is the deepest living *Truncatoflabellum* as well as the most geographically widespread.

### Truncatoflabellum inconstans

Taxon classificationAnimaliaScleractiniaFlabellidae

(Marenzeller, 1904)

Fig. 9D

Flabellum inconstans Marenzeller, 1904: 277-280, pl. 17, fig. 11a–h.—Boshoff 1981: 34–35.
Flabellum harmeri : Boshoff 1981: 35 (in part).
Truncatoflabellum inconstans : Cairns 1989b: 61.—Zibrowius and Gili 1990: 39 (comparison to other species).—Cairns and Keller 1993: 220 (listed).

#### Additional record.

AFR 985c, 34°47'S, 20°19'E, 80 m, 5.4.1948, 1, SAM .

#### Distribution.

Known only from off southern South Africa, 23-130 m.

#### Remarks.

It is tempting to include Zibrowius and Gili’s (1990) *Truncatoflabellum* sp. A form Walvis Ridge (1152 m) as an aberrant *Truncatoflabellum inconstans*, but as they say, their unique specimen has many fewer septa, a smaller basal scar, and is found much deeper than typical *Truncatoflabellum inconstans*. Their unidentified specimen is thus not assigned to a species.

Very rarely a pair of very small basal thecal spines may be present, but the species is considered to lack spines for the purpose of the key.

### Truncatoflabellum corbicula

Taxon classificationAnimaliaScleractiniaFlabellidae

(Tenison-Woods, 1880)

Fig. 9C

Flabellum corbicula Tenison-Woods, 1880: 13, figs 10a–b.—Squires 1958: 66 (lectotype designated).
Truncatoflabellum corbicula : Cairns 1989b: 61.

#### New record.

NZGS 1341, Wharekuri Greensand, Wharekuri, Waitaki Valley, New Zealand, S117/492, Duntroonian (Lower Oligocene), 1 specimen, USNM 67939.

#### Distribution.

Port Hills, Nelson, and Waitaki Valley, New Zealand (Duntroonian =Lower Oligocene).

#### Remarks.

The name *corbicula*, Latin for small basket, is treated as a noun in apposition and thus does not match gender with the genus.

### Truncatoflabellum truncum

Taxon classificationAnimaliaScleractiniaFlabellidae

(Cairns, 1982)

Fig. 10A

Flabellum truncum Cairns, 1982: 46, pl. 14, figs 5-8.
Truncatoflabellum truncum : Cairns 1994: 114.—Cairns et al. 2005: 17 (listed).—Cairns and Polonio 2013: 60 (listed).

#### Distribution.

Peru to southern Chile, Falkland Islands, 595–1896 m.

#### Remarks.

This species is known only from its original description.

### Truncatoflabellum trapezoideum

Taxon classificationAnimaliaScleractiniaFlabellidae

(Keller, 1981)

Flabellum trapezoideum Keller, 1981: 28, 31, pl. 1, figs 2a–b.
Truncatoflabellum trapezoideum : Cairns 1989b: Table 6; 1994: 79; 1995: 114.

#### Distribution.

Marcus-Necker Ridge, central North Pacific, 1630 m.

#### Remarks.

The species is known from only one specimen. It is very similar to *Truncatoflabellum truncum* Cairns, 1982 (see Key and Table 2). Nomenclaturally, this species is similar to *Flabellum trapezoidale* Osasco, 1895, a true *Flabellum* known only from the Pliocene of Italy.

### Truncatoflabellum formosum

Taxon classificationAnimaliaScleractiniaFlabellidae

Cairns, 1989b

Fig. 10B

Truncatoflabellum formosum Cairns, 1989b: 69–70, Table 6, (in part: not *Alb* 5137, 5162, 5483, 5484), pl. 35j–k, 36a–b (synonymy).—Cairns and Keller 1993: 264, 265, figs 10I, 11A.—Cairns 1994: 77, pl. 33 g–h.—Cairns and Zibrowius 1997: 169–170.—Cairns 2004: 309.
Truncatoflabellum sp. Cairns, 1989b: 73 (undescribed decameral).

#### Distribution.

Philippines, Indonesia, Japan, Korea Strait, New Caledonia, western Australia, southwest Indian Ocean, 42–933 m.

### Truncatoflabellum carinatum

Taxon classificationAnimaliaScleractiniaFlabellidae

Cairns, 1989b

Fig. 10C

?Flabellum variabile forma alta Gerth, 1921: 401, pl. 57, fig. 16.—Cairns 1989b: pl. 38f.
Flabellum rubrum : Yabe and Eguchi 1942a: 96–98 (in part: pl. 8, figs 6–12, 20).—Umbgrove 1950: 641, in part: pl. 81, figs 5–6.—Cairns 1989b: pl. 38d.
Truncatoflabellum carinatum Cairns, 1989b: 73–74, Table 6, pl. 38b–e (synonymy); 1994: 77–78, pl. 33j–k.
Flabellum transversale : Hu 1987: pl. 3, figs 1–2.
Flabellum rubrum stokesii : Hu 198: 150, in part: pl. 2, figs 12–14.

#### New records.

*Anton Bruun* 7, 372-J and L, 25°07'S, 34°34'E, 105–112 m, 9 specimens, USNM 1279595 and 127 9596. Plio-Pleistocene, Ryukyu Islands, Okinawa, 4 specimens, USNM 88445.

#### Distribution.

?Pliocene of Java (Gerth 1921); Pliocene of Ryukyu Islands (Yabe and Eguchi 1942b); Pliocene Taiwan (Hu 1987, 1988); Pleistocene (Java) (Umbgrove 1950); Holocene: South China Sea, Indonesia, off Mozambique, 30–274 m.

#### Remarks.

If Gerth’s (1921) forma *alta* is conspecific, it would have nomenclature priority as *Truncatoflabellum altum*.

### Truncatoflabellum gardineri

Taxon classificationAnimaliaScleractiniaFlabellidae

Cairns in Cairns & Keller, 1992

Fig. 10D

Truncatoflabellum gardineri Cairns in Cairns & Keller, 1993: 266–267, figs 11B–D.—Cairns 1994: 78–79, pl. 34a–b.

#### Distribution.

Off South Africa, Japan, 100–144 m.

### Truncatoflabellum arcuatum

Taxon classificationAnimaliaScleractiniaFlabellidae

Cairns, 1995

Fig. 11A

Truncatoflabellum arcuatum Cairns, 1995: 116, pl. 38g-i.

#### Distribution.

Norfolk and Kermadec Ridges, 350-364 m.

### Genus Blastotrochus

Taxon classificationAnimaliaScleractiniaFlabellidae

Milne Edwards & Haime, 1848

Blastotrochus Milne Edwards & Haime, 1848: 284–285.—Cairns 1989a: 645; 1989b: 74 (synonymy, discussion).—Cairns and Kitahara 2012: 14 (key to genus).
Flabellum (Blastotrochus) : Duncan 1884: 14.
Flabellum : Vaughan and Wells 1943: 226 (in part).—Wells 1956: F432 (in part).—Zibrowius 1974: 19–20 (in part: part of group 2).

#### Diagnosis.

Like *Truncatoflabellum*, but also producing asexual buds (anthoblasts) from thecal edges of anthocyathus. Thecal edges rounded, have a low edge angle, and bear one pair of basal edge spines.

#### Discussion.

The mode of asexual reproduction employed by *Blastotrochus*, described and illustrated by Cairns (1989a) as the anthoblast mode (also called bud shedding), differs slightly from transverse division of *Truncatoflabellum* by its potential to produce many more simultaneous clonemates from its thecal edges (instead of one at a time as with *Truncatoflabellum*), leading to a potentially exponential increase in clonemates instead of a gradual one. This was considered as a key innovation by Cairns (1989a), worthy of generic distinction from *Truncatoflabellum*. A second species was described in this genus, *Blastotrochus proliferus* d’Archiardi, 1866 (Miocene, Italy), but was reassigned to *Cladocora* (see Pfister 1980). *Blastotrochus* thus remains a monophyletic genus and has rarely been collected.

#### Distribution.

Philippines, Indonesia, 11-62 m.

#### Type species.

*Blastotrochus nutrix* Milne Edwards & Haime, 1848, by monotypy.

### Blastotrochus nutrix

Taxon classificationAnimaliaScleractiniaFlabellidae

Milne Edwards & Haime, 1848

Fig. 11B

Blastotrochus nutrix Milne Edwards & Haime, 1848: 284–285, pl. 8, Fig. 14.—Semper 1872: 238–241, pl. 16, figs 1–6.—Chevalier 1961: 379.—Cairns 1989a: 643, 645, fig. 1 (upper); 1989b: 74–75, pl. 38i-m, 39a–b (synonymy).—Cairns and Zibrowius 1997: 173–174.—Cairns and Kitahara 2012: pl. 23A–B.

#### Distribution.

As for the genus.

### Genus Placotrochides

Taxon classificationAnimaliaScleractiniaFlabellidae

Alcock, 1902

Placotrochides Alcock, 1902: 33.—Zibrowius 1974: 20, 23, 26.—Cairns 1989b: 78 (synonymy, discussion); 1995: 116; 2004: 307 (key to species).—Cairns and Kitahara 2012: 13 (key to genus).
Flabellum : Vaughan and Wells 1943: 226 (in part).

#### Diagnosis.

Asexual reproduction by apical transverse division of corallum, resulting in distal anthocyathus and basal anthocaulus. Corallum usually laterally compressed and subcylindrical, having a low edge angle; thecal edges rounded and do not bear spines or crests; calicular outline often asymmetrical. Columella absent of represented by a fusion of the lower, axial edges of the larger septa. Anthocaulus stereome-reinforced.

#### Discussion.

*Placotrochides* differs from *Truncatoflabellum* by having a non-spinose compressed-cylindrical corallum and a stereome-reinforced anthocaulus.

#### Distribution.

Western and central Pacific, southwestern Indian Ocean, northern and southwestern Atlantic, 80-1628 m.

#### Type species.

*Placotrochides scaphula* Alcock, 1902, by subsequent designation (Wells 1936).

### Key to the species of *Placotrochides* (characters pertain to the anthocyathus stage)

**Table:** 

1	GCD > 12 mm	***Placotrochides scaphula*** (Fig. 11C)
1’	GCD < 7 mm	**2**
2	S1>S2; GCD:LCD = 1.07–1.16 (close to circular)	***Placotrochides cylindrica*** (Fig. 12A)
2’	S1=S2; GCD:LCD = 1.19–2.0 (more elliptical)	**3**
3	GCD rarely greater than 3.5 mm; GCD:LCD = 1.6–2.0; Indo-West Pacific	***Placotrochides minuta*** (Fig. 11D)
3’	GCD about 5 mm; GCD:LCD = 1.2–1.5; amphi-Atlantic	***Placotrochides frustum*** (Fig. 12B)

### Placotrochides scaphula

Taxon classificationAnimaliaScleractiniaFlabellidae

Alcock, 1902

Fig. 11C

Placotrochides scaphula Alcock, 1902: 34, pl. 4, figs 32, 32a.—Cairns 1989b: 45, 78–79, pls. 40l, 41a–e (synonymy).—Cairns and Parker 1992: 48–49, figs 15h–i.—Cairns and Keller 1993: 272–273, figs 12D, G.—Cairns 1994: 79–80, pl. 34f–h; 1995: 116–117, pls. 38j, 39a.—Cairns and Zibrowius 1997: 174; 2004: 307.—Cairns and Kitahara 2012: pl. 20, figs N–O.
Flabellum elongatum Hu, 1987: 44, pl. 3, figs 4, 7–8 (also a junior homonym of *Flabellum elongatum* Milne Edwards & Haime, 1848: 275).

#### Distribution.

Plio-Pleistocene: southern Taiwan (Hu 1987). Holocene: off Japan, Philippines, Indonesia, New Caledonia, New Zealand, off Victoria and Queensland, Australia, southwest Indian Ocean, 80–1628 m.

#### Remarks.

A replacement name for junior primary homonym *Flabellum elongatum* Hu, 1987 is not necessary, as the senior homonym is considered to belong to *Truncatoflabellum* and Hu’s species to *Placotrochides*.

### Placotrochides cylindrica

Taxon classificationAnimaliaScleractiniaFlabellidae

Cairns, 2004

Fig. 12A

Placotrochides cylindrica Cairns, 2004: 305, 307 (key), figs 10B–D.

#### Distribution.

Known only from seamounts off northeastern Australia, 1117–1402 m.

### Placotrochides minuta

Taxon classificationAnimaliaScleractiniaFlabellidae

Cairns, 2004

Fig. 11D

Placotrochides sp. n. Feinstein & Cairns, 1998: 81, 83, fig. 10.
Placotrochides minuta Cairns, 2004: 305–307 (key), figs 10E–H.
Placotrochides minima : Cairns 2006: 52 (*lapsus calumni*).

#### Distribution.

Marion Plateau of Queensland, Indonesia, Hawaii, 119–458 m.

### Placotrochides frustum

Taxon classificationAnimaliaScleractiniaFlabellidae

Cairns, 1979

Fig. 12B

Placotrochides frusta Cairns, 1979: 152–153, pl. 29, figs 4–6, 8–9, map 43.
Placotrochides frustra : Zibrowius 1980: 159–161, pl. 81E-M.
Placotrochides frustum : Cairns 2004: 307 (key, nom. correct.).

#### New records.

CRYOS, *Balgim* CP85, 34°24'N, 7°39'W, 1378 m, 15 specimens, MNHN; *Professor Logachev* 37L 165, 16°54.014'N, 46°34.842'W, 2646–2705 m, 6 Mar 2015, 1, USNM 1295415, 1, IOM Moscow; *Professor Logachev* 37L 188, 17°08.470'N, 46°23.443'W, 2291–2327 m, 12 Mar 2015, 1, IOM Moscow.

#### Distribution.

Lesser Antilles, off northeastern Brazil, mid-Atlantic Ridge at latitude of Lesser Antilles, off Morocco, 497–2646 m.

#### Remarks.

The specimens reported herein from the mid-Atlantic Ridge are much larger than any previously reported, having a GCD up to 10.2 mm and a height of 13.9 mm, the calice having corresponding more septa, i.e., S1-2>S3>S4, 12:12:12, or 36 septa. The largest previously known specimen was only 5.0 mm in GCD and had 26 septa. They also represent a considerable depth range extension.

### Genus Placotrochus

Taxon classificationAnimaliaScleractiniaFlabellidae

Milne Edwards & Haime, 1848

Placotrochus Milne Edwards & Haime, 1848: 282.—Duncan 1884: 16 (in part: not fossil records).—Vaughan and Wells 1943 227 (in part: not fossil records).—Wells 1956: F432.—Zibrowius 1974: 21, 26.—Cairns 1989: 45, 75 (synonymy).—Cairns and Kitahara 2012: 13 (key to genus).

#### Diagnosis.

Asexual reproduction by apical transverse division of corallum, resulting in distal anthocyathus and basal anthocaulus. Corallum laterally compressed and fan shaped, having rounded thecal edges with one pair of basal thecal edge spines. Columella lamellar. Anthocaulus not stereome-reinforced.

#### Discussion.

Seven species of *Placotrochus* were described from the Australian Eocene-Miocene by Duncan (1864), Dennant (1899, 1903, 1904), and Tenison-Woods (1878a), but these species are not transversely dividing and thus should be assigned to a different genus (Cairns in prep.). *Placotrochus* is a monotypic genus.

#### Distribution.

Western Pacific, eastern Indian Ocean, 6–289 m.

#### Type species.

*Placotrochus laevis* Milne Edwards & Haime, 1848, by subsequent designation (Milne Edwards and Haime 1850: xviii).

### Placotrochus laevis

Taxon classificationAnimaliaScleractiniaFlabellidae

Milne Edwards & Haime, 1848

Fig. 12C

Placotrochus laevis Milne Edwards & Haime, 1848: 283, pl. 8, figs 15, 15a.—Semper 1872: 251–252, pl. 18, figs 11–13.—Bourne 1905: 200–201, pl. 1, fig. 5.—Cairns 1989b: 75–76, pl. 39c–g (synonymy).—Cairns and Zibrowius 1997: 175.—Cairns 2004: 307 (synonymy).—Cairns and Kitahara 2012: pl. 20, figs I–J.
Placotrochus candeanus Milne Edwards & Haime, 1848: 283–284.
Placotrochus pedicellatus Tenison-Woods, 1879: 134–135, pl. 13, figs 7, 7a.

#### New record.

*Alpha Helix* M-21: 8°45'S, 144°05.8'E, 55 m, 1 specimen, USNM 1130681.

#### Distribution.

As for genus.

### Genus Falcatoflabellum

Taxon classificationAnimaliaScleractiniaFlabellidae

Cairns, 1995

Falcatoflabellum Cairns, 1995: 117–118.—Cairns and Kitahara 2012: 14 (key to genus).

#### Diagnosis.

Asexual reproduction by apical transverse division of corallum, resulting in distal anthocyathus and basal anthocaulus. Corallum compressed-cylindrical, often slightly curved, with rounded thecal edges that lack spines and crests. Columella fascicular; paliform lobes occasionally present before S2. Anthocaulus unknown.

#### Discussion.

*Falcatoflabellum* is easily distinguished from all other flabellids by its fascicular columella and paliform lobes (P2). The genus is monotypic.

#### Distribution.

Kermadec Islands, 366-402 m.

#### Type species.

*Falcatoflabellum raoulensis* Cairns, 1995, by original designation.

### Falcatoflabellum raoulensis

Taxon classificationAnimaliaScleractiniaFlabellidae

Cairns, 1995

Fig. 12D

Falcatoflabellum raoulensis Cairns, 1995: 118, pl. 39b-g.—Cairns and Kitahara 2012: pl. 20, figs K–M.

#### Distribution.

As for genus.

#### Remarks.

Known only from the type series of 21 specimens from the type-locality.

## Supplementary Material

XML Treatment for Truncatoflabellum

XML Treatment for Truncatoflabellum phoenix

XML Treatment for Truncatoflabellum gippslandicum

XML Treatment for Truncatoflabellum victoriae

XML Treatment for Truncatoflabellum dens

XML Treatment for Truncatoflabellum zuluense

XML Treatment for Truncatoflabellum pusillum

XML Treatment for Truncatoflabellum angustum

XML Treatment for Truncatoflabellum angiostomum

XML Treatment for Truncatoflabellum macroeschara

XML Treatment for Truncatoflabellum veroni

XML Treatment for Truncatoflabellum gambierense

XML Treatment for Truncatoflabellum irregulare

XML Treatment for Truncatoflabellum incrustatum

XML Treatment for Truncatoflabellum sphenodeum

XML Treatment for Truncatoflabellum crassum

XML Treatment for Truncatoflabellum aculeatum

XML Treatment for Truncatoflabellum mortenseni

XML Treatment for Truncatoflabellum australiensis

XML Treatment for Truncatoflabellum candeanum

XML Treatment for Truncatoflabellum compressum

XML Treatment for Truncatoflabellum martensii

XML Treatment for Truncatoflabellum mozambiquensis

XML Treatment for Truncatoflabellum vigintifarium

XML Treatment for Truncatoflabellum spheniscus

XML Treatment for Truncatoflabellum cumingi

XML Treatment for Truncatoflabellum vanuatu

XML Treatment for Truncatoflabellum duncani

XML Treatment for Truncatoflabellum multispinosum

XML Treatment for Truncatoflabellum paripavoninum

XML Treatment for Truncatoflabellum stabile

XML Treatment for Truncatoflabellum inconstans

XML Treatment for Truncatoflabellum corbicula

XML Treatment for Truncatoflabellum truncum

XML Treatment for Truncatoflabellum trapezoideum

XML Treatment for Truncatoflabellum formosum

XML Treatment for Truncatoflabellum carinatum

XML Treatment for Truncatoflabellum gardineri

XML Treatment for Truncatoflabellum arcuatum

XML Treatment for Blastotrochus

XML Treatment for Blastotrochus nutrix

XML Treatment for Placotrochides

XML Treatment for Placotrochides scaphula

XML Treatment for Placotrochides cylindrica

XML Treatment for Placotrochides minuta

XML Treatment for Placotrochides frustum

XML Treatment for Placotrochus

XML Treatment for Placotrochus laevis

XML Treatment for Falcatoflabellum

XML Treatment for Falcatoflabellum raoulensis
